# Impaired myocellular Ca^2+^ cycling in protein phosphatase PP2A-B56α KO mice is normalized by β-adrenergic stimulation

**DOI:** 10.1016/j.jbc.2022.102362

**Published:** 2022-08-10

**Authors:** Dennis Glaser, Alexander Heinick, Julius R. Herting, Fabian Massing, Frank U. Müller, Paul Pauls, Timofey S. Rozhdestvensky, Jan S. Schulte, Matthias D. Seidl, Boris V. Skryabin, Frank Stümpel, Uwe Kirchhefer

**Affiliations:** 1 Institute of Pharmacology and Toxicology, University of Münster, Münster, Germany; 2 Department of Medicine, Core Facility Transgenic Animal and Genetic Engineering Models (TRAM), University of Münster, Münster, Germany

**Keywords:** PP2A-B56α, protein phosphorylation, myocellular Ca^2+^ handling, LTCC, β-adrenergic function, AAV, adeno-associated virus, cDNA, complementary DNA, CSQ, calsequestrin, HZ, heterozygous, ISO, isoprenaline, KO, homozygous, KSOM, potassium simplex optimized medium, LTCC, L-type Ca^2+^ current, NCX, sodium-calcium exchanger, PLN, phospholamban, SL, sarcomere length, SR, sarcoplasmic reticulum, WT, wild-type

## Abstract

The activity of protein phosphatase 2A (PP2A) is determined by the expression and localization of the regulatory B-subunits. PP2A-B56α is the dominant isoform of the B′-family in the heart. Its role in regulating the cardiac response to β-adrenergic stimulation is not yet fully understood. We therefore generated mice deficient in B56α to test the functional cardiac effects in response to catecholamine administration *versus* corresponding WT mice. We found the decrease in basal PP2A activity in hearts of KO mice was accompanied by a counter-regulatory increase in the expression of B′ subunits (β and γ) and higher phosphorylation of sarcoplasmic reticulum Ca^2+^ regulatory and myofilament proteins. The higher phosphorylation levels were associated with enhanced intraventricular pressure and relaxation in catheterized KO mice. In contrast, at the cellular level, we detected depressed Ca^2+^ transient and sarcomere shortening parameters in KO mice at basal conditions. Consistently, the peak amplitude of the L-type Ca^2+^ current was reduced and the inactivation kinetics of *I*_CaL_ were prolonged in KO cardiomyocytes. However, we show β-adrenergic stimulation resulted in a comparable peak amplitude of Ca^2+^ transients and myocellular contraction between KO and WT cardiomyocytes. Therefore, we propose higher isoprenaline-induced Ca^2+^ spark frequencies might facilitate the normalized Ca^2+^ signaling in KO cardiomyocytes. In addition, the application of isoprenaline was associated with unchanged L-type Ca^2+^ current parameters between both groups. Our data suggest an important influence of PP2A-B56α on the regulation of Ca^2+^ signaling and contractility in response to β-adrenergic stimulation in the myocardium.

The serine/threonine protein phosphatase (PP) 2A (PP2A) belongs to the major superfamily of PPs. PP2A exists in the cell as a heterodimer consisting of a structural and catalytic subunit or as a heterotrimer that includes an additional regulatory subunit (1). The regulatory B-type subunits modulate PP2A activity and determine the substrate specificity of the associated catalytic subunit *via* a cell- and/or tissue-specific expression (2, 3). Of the regulatory subunits, the members of the B′ family have been the best studied to date. B56α (*Ppp2r5a* gene) is a member of the B′ family and is the most highly expressed isoform in the heart (4, 5). The decreased expression and activity of B56α in failing human hearts (6) suggests a prominent role in cardiac function. In cardiomyocytes, PP2A-B56α associates with ion channels, ion pumps, and Ca^2+^ regulatory and myofilament proteins, regulating action potential, myocellular Ca^2+^ signaling, and contractility (7, 8, 9, 10). The functional role of PP2A-B56α on these targets was previously studied by use of genetically modified expression models. Our group demonstrated for the first time that cardiac-specific overexpression of B56α led to a parallel increase in PP2A activity (11). This was associated with a lower phosphorylation of contractile proteins and an improved myofilament Ca^2+^ sensitivity resulting in an increased contractility. However, the β-adrenergic effect was attenuated in transgenic hearts. In contrast, AAV-associated overexpression of B56α in mouse hearts resulted in a decreased PP2A activity and an increased phosphorylation of RyR2 (12). The maximal chronotropic effect of isoprenaline (ISO) was increased. The findings of these contrary studies are complicated by the results of mouse models with deletion of B56α. The global KO in mice resulted in an increased PP2A activity (12). The decreased phosphorylation of RyR2 was accompanied by a reduced Ca^2+^ spark frequency after β-adrenergic stimulation (12). In contrast, global deletion of B56α in a gene trap model resulted in a decrease in PP2A activity that was associated with a diminished RyR2 phosphorylation (13). Echocardiography showed an attenuated inotropy after an acute β-adrenergic stimulation. Although all studies on genetically modified PP2A-B56α mouse models demonstrated a high relevance of this regulatory subunit in affecting cardiac Ca^2+^ homeostasis and force development, the data are highly controversial and phenotypically and functionally not fully understood. A common feature of all studies seems to be that the physiological effects are independent of B56α but more a direct consequence of the PP2A activity. In addition, it cannot be derived from these animal studies whether the altered expression of B56α observed in human heart failure of various etiologies (5, 6) is beneficial or even detrimental for the maintenance of contractile function and the β-adrenergic response.

This study, therefore, aims at more comprehensively studying the functional consequences of a global silencing of the *Ppp2r5a* gene on cardiac contractility. Moreover, this is the first study to evaluate in detail the functional effects of a PP2A-B56α KO on myocellular Ca^2+^ signaling in response to β-adrenergic stimulation by use of electrophysiological experiments.

## Results

### Reduced PP2A activity is associated with an enhanced protein expression of other B′ family members in KO hearts

**Figure 1:**
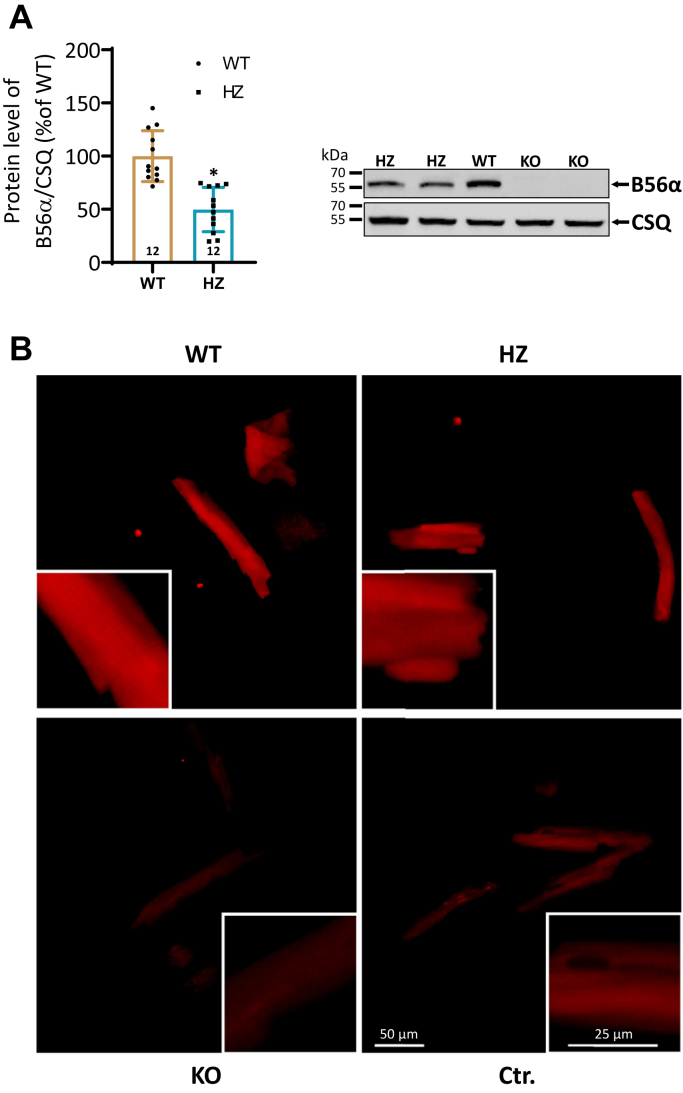
**Expression and localization of B56α in the heart.***A*, quantification of the B56α protein expression in hearts of WT and heterozygous (HZ) mice (*left panel*; ∗*p* < 0.05 *versus* WT; *t* test; N = hearts). A representative immunoblot of all genotypes is shown (*right panel*). Individual samples were used. Calsequestrin (CSQ) served as a reference protein. Molecular weight markers were included on the left side of the blots. *B*, photomicrographs showing detailed analysis of isolated cardiomyocytes from WT, HZ, and KO mouse hearts by confocal microscopy. Images were taken with the same microscope settings for all genotypes. Red fluorescence staining represents B56α. The control (Ctr.) was performed without the primary antibody raised against B56α. Note the loss of the B56α signal in KO cardiomyocytes. Detailed analysis (small insets) revealed both a diffuse and sarcomeric pattern in the distribution of B56α in WT and HZ suggesting a cytosolic and myofilamental localization.

**Figure 2:**
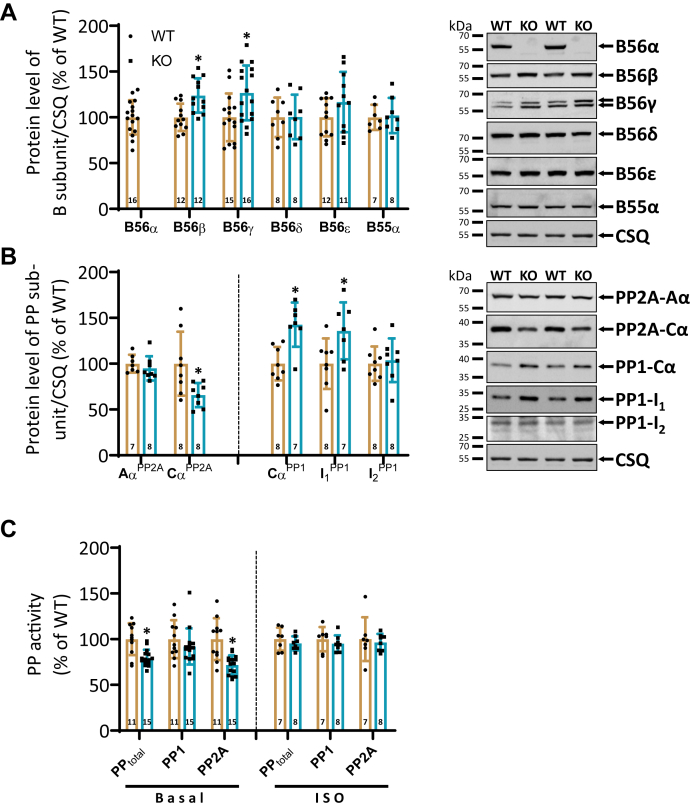
**Loss of B56α is accompanied by a lower PP2A activity.***A*, quantification of the protein expression of regulatory B and B′ subunits in hearts of WT and homozygous (KO) mice (*left panel*; ∗*p* < 0.05 *versus* WT; *t* test; N = hearts). Shown are representative Western blots (*right panel*). Calsequestrin (CSQ) served as a reference protein. One set of samples was available for detection of proteins that was probed with the CSQ antibody and then used for both Figures 2 and 4. Molecular weight markers were included on the left side of the blots. *B*, protein expression of structural (PP2A-Aα), catalytic (PP1-Cα & PP2A-Cα), and inhibitory (PP1-I_1_ and -I_2_) subunits in cardiac homogenates of WT and KO mice (*left panel*; ∗*p* < 0.05 *versus* WT; *t* test; N = hearts). Representative immunoblots are depicted at the right-hand side. CSQ served as a reference protein. The same CSQ loading control was used for all other blots. Molecular weight markers were included on the left side of the blots. *C*, PP activity was determined in heart samples of WT and KO mice under basal conditions and after acute administration with isoprenaline (ISO). PP1 and PP2A activities were obtained by incubation of heart samples with 3 nM okadaic acid. At this concentration, a complete inhibition of PP2A is achieved, leaving PP1 activity unaffected. PP activities in WT heart homogenates were set to 100% (∗*p* < 0.05 *versus* WT; *t* test; N = hearts).

First, the expression level of B56α in the heart was tested by Western blotting. The expression was reduced by 49% in heterozygous (HZ) mice (Fig. 1*A*). As expected, B56α was undetectable in homozygous KO animals. The loss of B56α was also evident in immunofluorescence stainings of KO cardiomyocytes (Fig. 1*B*). Analysis of B56α distribution in isolated cardiomyocytes by confocal laser microscopy revealed both a cytosolic and sarcomeric pattern in WT and HZ, suggesting that a reduction in the expression of B56α has no effect on its localization (Fig. 1*B*). Deletion of B56α was evident in all tissues tested (Fig. S1*A*). Only homozygous animals were used for further experiments. Deletion of B56α was associated with an increased expression of regulatory subunits of the B′ family (Fig. 2*A*). B56β expression was increased by 24% and that of B56γ by 27%. Other subunits of this gene family as well as B55α from the B family remained unchanged. B56β and B56γ are 69.6% and 66.2% identical in sequence to B56α, respectively, and show a comparable cellular localization (Fig. S2). Thus, the increased expression of both subunits may counteract the loss of B56α. Deletion of B56α was accompanied by a 34% reduction in the catalytic subunit of PP2A, whereas the expression of PP1 was increased by 43% (Fig. 2*B*). However, the concomitant upregulation of the inhibitor-1 of PP1 in KO hearts (Fig. 2*B*) may limit this increase in PP1 expression. The catalytic subunit of PP2A was also detected in immunofluorescence stainings (Fig. S1*B*). The decrease in expression of the catalytic subunit of PP2A in KO was accompanied by a decreased total PP and PP2A activity (by 22% and 30%, respectively, Fig. 2*C*). Moreover, the effect of increasing concentrations of okadaic acid on PP activity was investigated. The inhibition curve showed no differences between the 2 genotypes (Fig. S3). In contrast, total PP and PP2A activity were unchanged in ISO-stimulated heart preparations between both groups (Fig. 2*C*).

### Unchanged cardiac morphology in KO mice

In a previous work, we showed that the expression of B56α and the catalytic subunit of PP2A is decreased in left ventricles of insufficient human hearts (6). Therefore, it was investigated whether deletion of B56α is associated with the development of cardiac hypertrophy. For this purpose, histological preparations of extracted hearts of both genotypes were examined (Fig. S4*A*). The heart to body weight ratio was unchanged between KO and WT mice (Fig. S4*B*). Evaluation of Masson-Goldner stainings exhibited an unchanged collagen content in KO hearts (Fig. S4*B*). Marker proteins for hypertrophy and/or fibrosis (*e.g*., BNP) were also unchanged at the mRNA level between KO and WT (Fig. S4*C*).

### Increased intraventricular pressure and relaxation at basal conditions in catheterized KO mice

**Figure 3:**
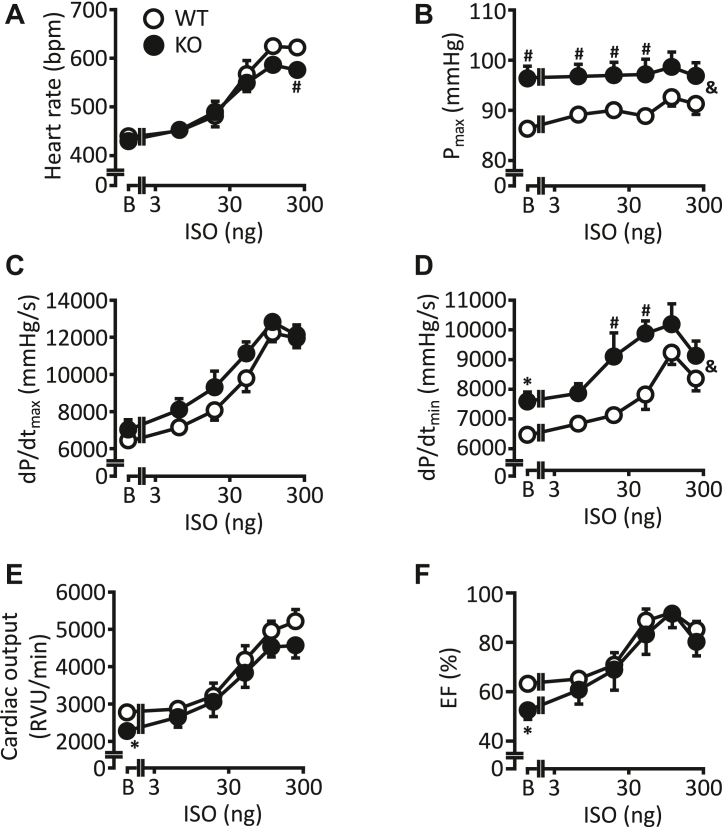
**Improved basal left-ventricular pressure and relaxation in catheterized KO mice.** Shown are the contractile data in anaesthetized left-ventricular catheterized WT and KO mice in the absence and presence of increasing doses of isoprenaline (ISO). *A*, heart rate in beats per minute. *B*, maximum left-ventricular pressure in mmHg. *C*, rate of contraction (dP/dt_max_) in mmHg/s. *D*, rate of relaxation (dP/dt_min_) in mmHg/s. *E*, cardiac output in RVU/min. *F*, ejection fraction in percentage (∗*p* < 0.05 *versus* WT: *t* test; ^&^*p* < 0.05 *versus* WT: two-way ANOVA; ^#^*p* < 0.05 *versus* WT: Holm–Sidak posthoc test; N = 7 WT mice; N = 8 KO mice).

The functional effects of B56α deletion on cardiac function were first tested in anesthetized animals by catheterization of the carotid artery. At the beginning of the experiment, aortic pressure was determined and was comparable between KO and WT animals (85.7 ± 7.6 *versus* 86.0 ± 4.8 mmHg, respectively). Spontaneous heart rate was also comparable between both genotypes under basal conditions (Fig. 3*A*). Maximal doses of ISO resulted in a smaller increase in heart rates in KO animals. Left ventricular pressure (P_max_) was already increased under basal conditions in KO mice but was not affected under β-adrenergic stimulation (Fig. 3*B*). The rate of left ventricular pressure increase (dP/dt_max_) was unchanged under basal conditions and after application of ISO (Fig. 3*C*). The rate of left ventricular pressure decline (dP/dt_min_) was accelerated under basal conditions and after administration of intermediate doses of ISO in KO compared to corresponding WT animals (Fig. 3*D*). This was also accompanied by an increased half-maximal ISO effect in KO compared to corresponding WT mice (−9343 ± 353 *versus* −7920 ± 170 mmHg/s, respectively, *p* < 0.05). However, the calculated ED_50_ of ISO as a measure of potency was not different between KO and WT (log[ED_50_] 1.38 ± 0.15 *versus* 1.58 ± 0.14, respectively, nonsignificant). For the development of left ventricular pressure increase, an unchanged EC_50_ value was also obtained. Cardiac output (Fig. 3*E*) and ejection fraction (Fig. 3*F*) were decreased under basal conditions in KO animals. For example, the ejection fraction was 52.5% in KO animals, which is below a value of 55%, which is considered as a normal function (14). However, β-adrenergic stimulation normalized the depressed basal parameters.

### Increased phosphorylation state of cardiac regulatory proteins in KO hearts

**Figure 4:**
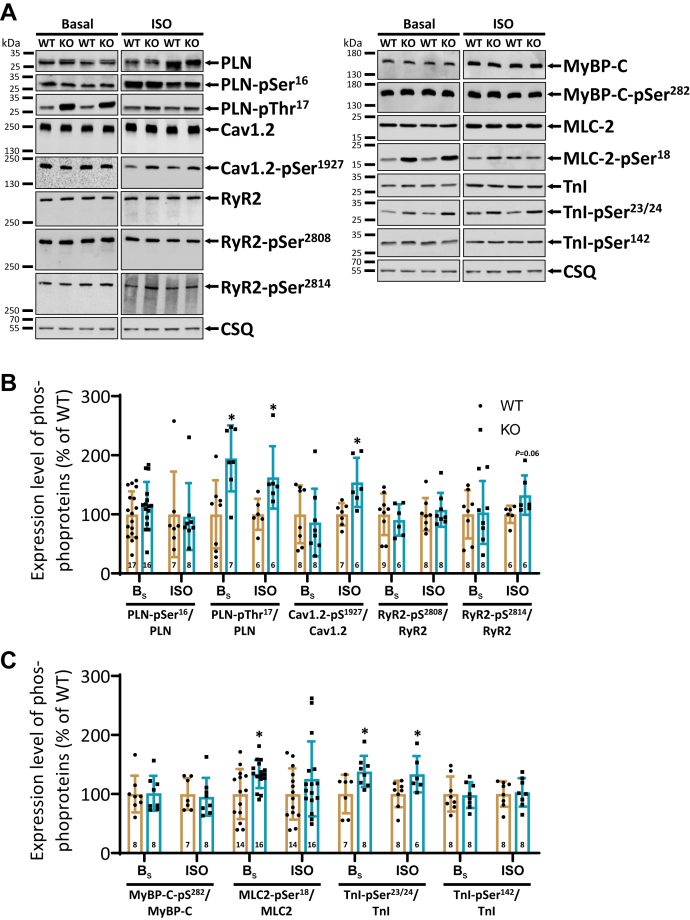
**Increased phosphorylation levels of Ca**^**2+**^**regulatory and myofilament proteins in KO hearts.***A*, representative immunoblots of cardiac Ca^2+^ regulatory and contractile proteins in WT and KO mice. Protein phosphorylation was measured in the absence (basal) and presence of isoprenaline (ISO). Calsequestrin (CSQ) served as a reference protein. One set of samples was available for detection of proteins that was probed with the CSQ antibody and then used for both Figures 2 and 4. Molecular weight markers were included on the left side of the blots. *B*, quantification of protein phosphorylation of sarcoplasmic reticulum (PLN, phospholamban; RyR2, ryanodine receptor type 2) and sarcolemmal proteins (Cav1.2, L-type Ca^2+^ channel) at basal (B_s_) and β-adrenergic stimulated (ISO) conditions. Normalization of phosphorylation levels was performed to the nonphosphorylated individual protein. Subsequently, the protein was adjusted to WT levels both under basal and stimulated conditions. *C*, quantification of phosphorylated myofilament proteins (MyBP-C, myosin-binding protein C; MLC2, myosin light chain 2; TnI, troponin inhibitor) (∗*p* < 0.05 *versus* WT; *t* test; N = hearts).

To investigate whether the hastened cardiac contraction and relaxation in KO animals were accompanied by parallel changes at the phosphorylation level of Ca^2+^ regulatory and contractile proteins, Western blot analyses were performed using cardiac homogenates from untreated and ISO-stimulated spontaneously beating mice. Representative immunoblots of the proteins studied are shown in Figure 4*A*. In line with a decreased PP2A activity in KO hearts, the basal phosphorylation state of phospholamban (PLN) on threonine-17 was increased by 95%, of the troponin inhibitor on serine-23/24 by 38%, and of MLC2 on serine-18 by 30% compared to corresponding WT hearts (Fig. 4, *B* and *C*). The degree of phosphorylation was also increased after administration of ISO for PLN-Thr^17^ and TnI-Ser^23/24^ in KO compared to corresponding WT. Moreover, the phosphorylation of the L-type Ca^2+^ channel on serine-1927 was increased only after β-adrenergic stimulation in KO but not under basal nonstimulated conditions compared to corresponding WT samples. A comparable effect was also detected for the phosphorylation of the ryanodine receptor on serine-2814. This amino acid is exclusively phosphorylated by Ca^2+^/calmodulin-dependent protein kinase II (CaMKII). The phosphorylation level was also unchanged after administration of ISO between both groups (*p* = 0.06). Basal expression and phosphorylation of other sarcoplasmic reticulum (SR) Ca^2+^ regulatory and myofilament proteins were unchanged between both genotypes (Fig. 4, *B* and *C*, Table 1).

**Table 1:** Expression of cardiac regulatory proteins

Name	WT (%)	KO (%)
Cav1.2	100.0 ± 6.0 (N = 16)	96.3 ± 4.5 (N = 16)
SERCA2a	100.0 ± 6.6 (N = 8)	108.9 ± 12.6 (N = 8)
Phospholamban	100.0 ± 7.8 (N = 16)	108.6 ± 8.7 (N = 16)
Ryanodine receptor type 2	100.0 ± 9.2 (N = 8)	98.5 ± 8.8 (N = 8)
Calsequestrin	100.0 ± 3.6 (N = 16)	97.4 ± 2.9 (N = 16)
Junctin	100.0 ± 12.1 (N = 7)	104.0 ± 13.5 (N = 8)
Triadin	100.0 ± 11.3 (N = 8)	99.8 ± 11.0 (N = 8)
NCX	100.0 ± 11.7 (N = 8)	94.8 ± 6.9 (N = 8)
Troponin inhibitor	100.0 ± 6.5 (N = 7)	103.8 ± 10.8 (N = 8)
Myosin light chain 2	100.0 ± 6.6 (N = 8)	102.7 ± 8.4 (N = 8)
Myosin-binding protein C	100.0 ± 7.8 (N = 8)	96.5 ± 6.1 (N = 8)

Levels of cardiac Ca^2+^ regulatory and myofilament proteins in homogenates of WT and KO mouse hearts were measured after scanning ECL-labeled immunoblots in a ChemiDoc XRS system (N = mice).

### Impaired basal myocellular contraction and Ca^2+^ transients but enhanced β-adrenergic response in KO cardiomyocytes

**Figure 5:**
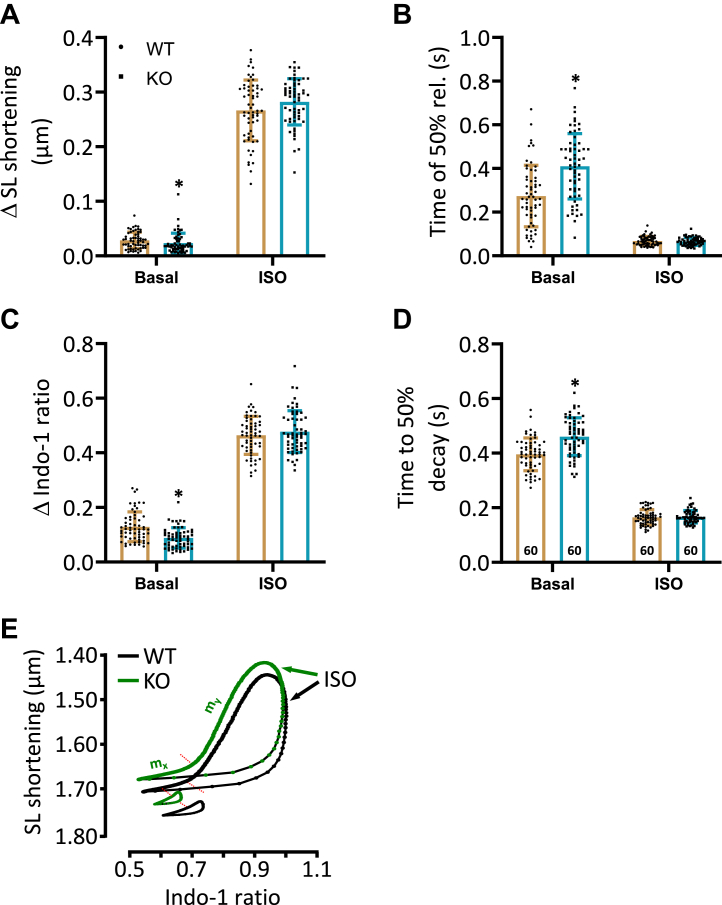
**Depressed contraction and Ca**^**2+**^**cycling but improved β-adrenergic response in KO cardiomyocytes.** Myocellular contraction and Ca^2+^ transient parameters were determined in electrically stimulated (0.5 Hz) cardiomyocytes of WT and KO mice under basal conditions and after stimulation with 1 μM isoprenaline (ISO). *A*, quantification of maximum sarcomere length (SL) shortening (ΔSL). *B*, quantification of the time to 50% relengthening. *C*, peak amplitude of Ca^2+^ transients. *D*, shown are the summarized data of the time to 50% decay of the Indo-1 signal (∗*p* < 0.05 *versus* WT: *t* test; n = cardiomyocytes (stated in the columns) of N = 6 WT hearts and N = 6 KO hearts). *E*, basal and ISO-stimulated Indo-1 ratio and SL were correlated. The slow (m_x_) and fast (m_y_) slopes (see Table 2) of this relation can be used as indicators of the myofilament Ca^2+^ sensitivity (Basal: n = 109 cardiomyocytes of 11 WT hearts and n = 123 cardiomyocytes of N = 12 KO hearts; ISO: n = 60 cardiomyocytes of N = 6 WT hearts and n = 60 cardiomyocytes of N = 6 KO hearts).

Next, we tested whether contractile effects observed in the living animal were associated with corresponding changes at the single cell level. For this purpose, sarcomere length (SL) shortening and Ca^2+^ transients were measured in electrically stimulated (0.5 Hz) isolated cardiomyocytes of both genotypes. To our surprise, a reduced maximum SL shortening was observed under basal conditions in KO compared to corresponding WT cardiomyocytes (Fig. 5*A*). However, the percentage increase under β-adrenergic stimulation was higher in KO compared to corresponding WT cells (1198 ± 48 *versus* 937 ± 50%, respectively, *p* < 0.05) resulting in a comparable absolute myocellular contraction between both groups (Fig. 5*A*). Moreover, the decay kinetics of myocyte contraction were analyzed. The time to 50% relengthening was prolonged in KO compared to corresponding WT cardiomyocytes under basal conditions (Fig. 5*B*). The unchanged relaxation parameter under administration of 1 μM ISO suggests an enhanced β-adrenergic effect in KO compared to corresponding WT cells (Fig. 5*B*). The changes in SL shortening under basal conditions and after stimulation with ISO were accompanied by parallel effects of Ca^2+^ transient parameters. The peak amplitude of Ca^2+^ transients was decreased under basal conditions in KO compared to corresponding WT myocytes (Fig. 5*C*). The application of catecholamines resulted in a comparable peak amplitude in both groups, suggesting an increased β-adrenergic effect in KO compared to corresponding WT cardiomyocytes (542 ± 26 *versus* 364 ± 13%, respectively, *p* < 0.05, *t* test). The half-maximum decay of Ca^2+^ transients was prolonged under nonstimulated conditions in KO compared to corresponding WT cardiomyocytes (Fig. 5*D*). However, the application of ISO was associated with comparable decay kinetics in both genotypes (Fig. 5*D*). The parallel changes in myocellular Ca^2+^ levels and contraction were reflected by an unchanged Ca^2+^ sensitivity of myofilaments in the presence of ISO (Fig. 5*E*, Table 2). This assumption is based on the unchanged slopes between KO and WT cells. In contrast, the slow (m_x_) component of the slope was increased under basal conditions along with a shift of the basal phase plane diagram upward and to the left in KO compared to corresponding WT cells, suggesting a higher myofilament Ca^2+^ sensitivity (15) that may compensate for the lower Ca^2+^ levels in KO cardiomyocytes.

**Table 2:** Slow and fast components of slopes derived from Indo-1 ratio - SL loops

Slope	WT	KO
m_x_, basal	0.19 ± 0.04	0.26 ± 0.01∗
m_y_, basal	0.39 ± 0.04	0.45 ± 0.12
m_x_, ISO	0.18 ± 0.03	0.17 ± 0.01^+^
m_y_, ISO	1.48 ± 0.14^+^	1.50 ± 0.08^+^

The slow (m_x_) and fast (m_y_) slopes can be used to estimate the Ca^2+^ sensitivity of myofilaments (∗*p* < 0.05 *versus* WT: *t* test; ^+^*p* < 0.05 *versus* basal: *t* test).

### Higher Ca^2+^ spark frequencies under application of ISO in KO cells

**Figure 6:**
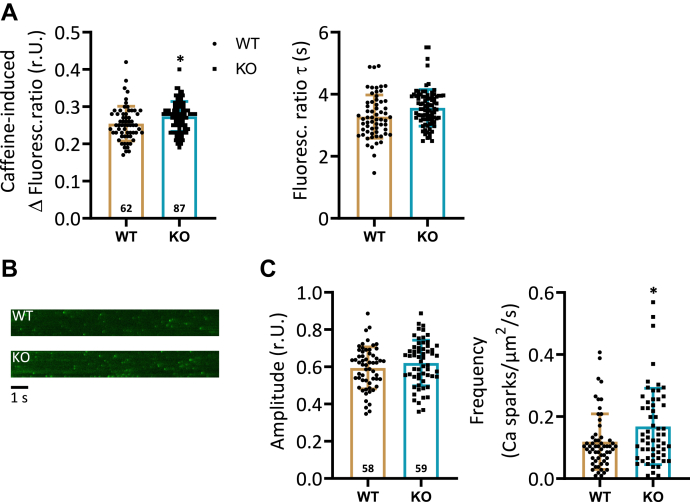
**Higher Ca**^**2+**^**spark frequencies after application of isoprenaline in KO cardiomyocytes.** Intracellular Ca^2+^ signaling was investigated in more detail by measurement of SR Ca^2+^ load and Ca^2+^ spark characteristics. *A*, shown are the maximum amplitude (*left panel*) and decay kinetics (*right panel*) of caffeine-induced Ca^2+^ transients in WT and KO cardiomyocytes (∗*p* < 0.05 *versus* WT; *t* test; n = cardiomyocytes (stated in the columns) of N = 6 WT hearts and N = 8 KO hearts). *B*, illustration of characteristic X-T scans of WT and KO cardiomyocytes after 1 Hz prestimulation and application of 1 μM isoprenaline detecting Ca^2+^ sparks. *C*, quantification of data for Ca^2+^ sparks amplitude (*left panel*) and frequency (CaSpF, *right panel*) in cells of both genotypes (∗*p* < 0.05 *versus* WT: *t* test; n = cardiomyocytes (stated in the columns) of N = 6 WT hearts and N = 6 KO hearts). SR, sarcoplasmic reticulum.

The influence of SR Ca^2+^ handling parameters on the observed impaired cardiomyocyte contractile and Ca^2+^ cycling properties was analyzed by measurement of caffeine-induced Ca^2+^ transients and confocal microscopy scans. A reduced SR Ca^2+^ content does not appear to be responsible for the reduced basal peak amplitude of Ca^2+^ transients because this was slightly increased by 8% in KO compared to corresponding WT cardiomyocytes (Fig. 6*A*). This moderate increase cannot be explained by an altered expression of SR Ca^2+^ regulatory proteins (Table 1). Phosphorylation levels of cardiac Ca^2+^ regulatory proteins were measured in tissue of spontaneously beating hearts in intact animals and may therefore not reflect the phosphorylation state in isolated cardiomyocytes. The decay kinetics of caffeine-induced Ca^2+^ transients were unchanged between KO and WT (Fig. 6*A*), suggesting a normal function of the sodium-calcium exchanger (NCX). The same was true for the protein expression of the NCX between both genotypes (Table 1).

The enhanced response to ISO in measurements of SL shortening and Ca^2+^ transients suggests an augmented β-adrenergic effect in KO. Corresponding changes in the phosphorylation state of SR Ca^2+^ regulatory proteins (Fig. 4*B*), taking into account a limited transferability of data from the whole animal to the single cell level, cannot in any case be held responsible for this effect. We therefore investigated whether an altered opening probability of the RyRs in the KO cardiomyocytes may account for the higher β-adrenergic response. The latter was determined by detection of Ca^2+^ spark characteristics in isolated cardiomyocytes. Cells were loaded with Fluo-4 and stimulated with 1 μM ISO at 1 Hz. During a stimulation pause of 12 s, X-T scans were recorded. Representative X-T scans of a WT and KO cardiomyocyte are shown in Figure 6*B*. The Ca^2+^ spark frequency was increased by 42% in KO compared to corresponding WT cells, whereas the Ca^2+^ spark amplitude remained unchanged (Fig. 6*C*).

### Depressed L-type Ca^2+^ current is normalized by application of ISO in KO cardiomyocytes

**Figure 7:**
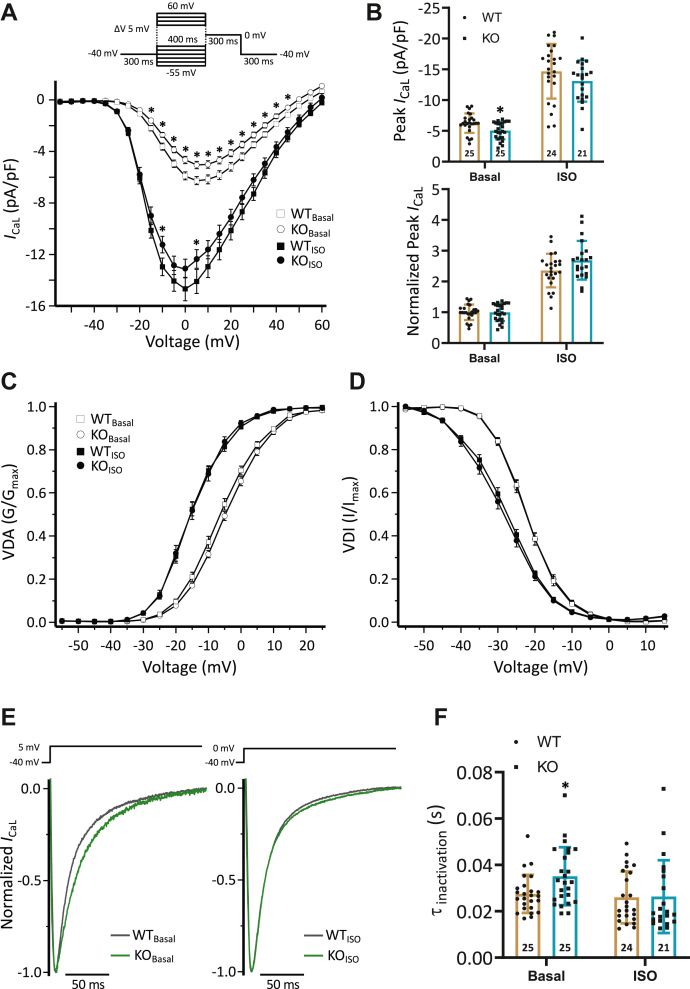
**Reduced *I***_**CaL**_**current densities are normalized by β-adrenergic stimulation in KO cardiomyocytes.** The effects of ISO on LTCC current parameters were determined in ventricular myocytes. *A*, current-voltage relations of L-type Ca^2+^ currents (*I*_CaL_) were measured in KO and corresponding WT cardiomyocytes in the absence (Basal) and presence of 1 μM ISO (∗*p* < 0.05 *versus* WT: 2-way RM ANOVA; n = cardiomyocytes/N = mice, see Table 3). *B*, bar graphs show the summarized maximum (*upper panel*) and normalized (*lower panel*) *I*_CaL_ current densities in the 4 groups (∗*p* < 0.05 *versus* WT: *t* test; n = cardiomyocytes (stated in the column) of N = mice, see Table 3). *C*, steady-state activation of *I*_CaL_ (VDA, voltage-dependent activation) in basal and ISO-treated WT and KO cardiomyocytes show the voltage dependence of normalized conductance (G) to G_max_. Symbols represent the measured values fitted with Boltzmann function represented. *D*, shown is the steady-state inactivation of *I*_CaL_ (VDI, voltage-dependent inactivation) in all 4 groups. *E*, shown are representative traces of steady-state *I*_CaL_, normalized to peak, measured in the absence (*left panel*) and presence (*right panel*) of ISO in WT (*gray*) and KO (*green*) cardiomyocytes. *F*, bar graphs show the time constants (τ_inact_) calculated from monoexponential fit of the *I*_CaL_ current decay (∗*p* < 0.05 *versus* WT: Mann–Whitney U-test; n = cardiomyocytes (stated in the column) of N = mice, see Table 3). ISO, isoprenaline; LTCC, L-type Ca^2+^ current.

To test whether the diminished peak amplitude of Ca^2+^ transients in KO cardiomyocytes is due to a corresponding modulation of L-type Ca^2+^ current (LTCC), we measured the LTCC (*I*_CaL_) in ventricular myocytes of both genotypes in the absence and presence of isoprenaline (ISO). The current-voltage relation of *I*_CaL_ densities was reduced in KO compared to corresponding WT cardiomyocytes under basal conditions (Fig. 7*A*). The maximum current density was decreased by 19% in KO compared to corresponding WT cardiomyocytes (Fig. 7*B*). In contrast to basal conditions, current-voltage relations of *I*_CaL_ densities were not different between KO and WT cardiomyocytes treated with 1 μM ISO (Fig. 7*A*). The maximum *I*_CaL_ density was increased in both genotypes in the presence of ISO, by 169% in KO and by 136% in WT (Fig. 7*B*), resulting, in summary, in a higher β-adrenergic response in KO cells (*p* = 0.062). The parameters calculated from the voltage-dependent activation and inactivation curves are presented in Table 3. Voltage-dependent activation (Fig. 7*C*) and voltage-dependent inactivation (Fig. 7*D*) were not different between both groups under basal conditions. ISO shifted both curves in the same direction to a comparable extent. For example, the voltage of half-maximal activation (V_1/2a_) was shifted by −10.7 ± 0.8 mV in KO and by −9.0 ± 0.6 mV in WT (*p* = 0.082). Representative *I*_CaL_ traces, normalized to peak, in the absence and presence of ISO of KO and WT ventricular myocytes are depicted in Figure 7*E*. At basal conditions, *I*_CaL_ inactivation was prolonged by 28% in KO compared to corresponding WT cardiomyocytes (Fig. 7*F*). However, the application of ISO caused a stronger effect in KO cells. The inactivation time was hastened by 25% in KO but only by 5% in WT (Fig. 7*F*). As a result, *I*_CaL_ integral was comparable between groups during ISO application (0.42 ± 0.03 *versus* 0.48 ± 0.03 pA∗s/pF, respectively, nonsignificant), whereas it was reduced by 21% in KO compared to corresponding WT cardiomyocytes under basal conditions (0.19 ± 0.01 *versus* 0.24 ± 0.01 pA∗s/pF, respectively, *p* < 0.05, *t* test, 25/5 = cardiomyocytes/mice each group).

**Table 3:** Parameters of LTCC activation and inactivation in the absence and presence of isoprenaline

Genotype	WT	WT	KO	KO
1 μM ISO	-	+	-	+
Activation				
n/N	25/5	24/5	24/5	21/5
V_1/2_ (mV)	−6.2 ± 0.7	−15.2 ± 0.8^+^	−4.5 ± 0.6	−14.9 ± 0.8^+^
Inactivation				
V_1/2_ (mV)	−22.2 ± 0.5	−28.2 ± 0.7^+^	−22.2 ± 0.6	−29.7 ± 1.0^+^
*I*_CaL_ kinetics				
n/N	25/5	24/5	25/5	21/5
τ_inact_ (ms)	27.5±1.6	26.0 ± 2.3^+^	35.1 ± 2.5∗	26.3 ± 3.42^+^

V_1/2_: potential of half-maximal activation and inactivation, τ_inact_: time constants of inactivation during a depolarizing pulse to 5 mV (basal) or 0 mV (ISO) calculated from exponential fit (^+^*p* < 0.05 Basal: *t* test; ∗*p* < 0.05 *versus* WT: Mann–Whitney U-test, n/N = cardiomyocytes/mice).

### Increased stimulation frequencies led to improved Ca^2+^ cycling and contractile parameters in KO cardiomyocytes

**Figure 8:**
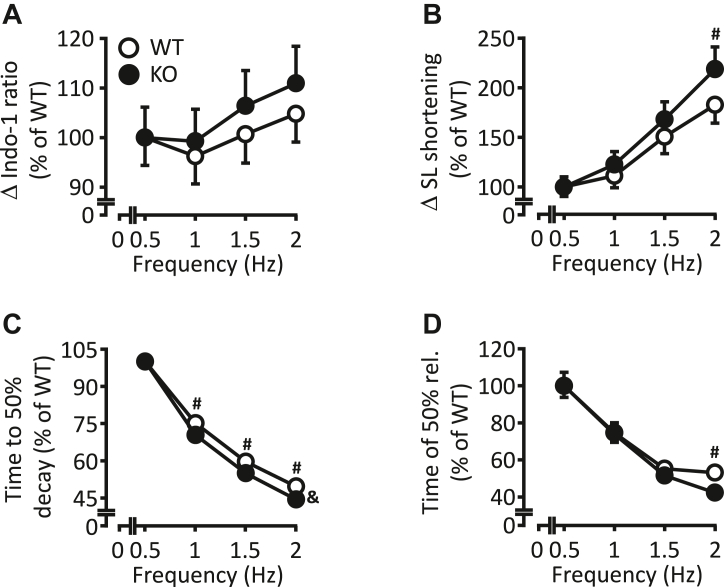
**Augmented contractile response to increasing stimulation frequencies in KO cardiomyocytes.** Graphical representation of Ca^2+^ transients and myocellular contraction under the influence of increasing stimulation frequencies in WT and KO cardiomyocytes. Data, collected at 0.5 Hz, were set to 100% for both genotypes. The following parameters were determined: *A*, the peak amplitude of Ca^2+^ transients. *B*, the maximum sarcomere length shortening. *C*, the time to 50% decay of Ca^2+^ transients. *D*, the time to 50% relengthening (^&^*p* < 0.05 *versus* WT: two-way ANOVA; ^#^*p* < 0.05 *versus* WT: Holm–Sidak post hoc test; n = 49 cardiomyocytes of N = 5 WT hearts and n = 63 cardiomyocytes of N = 6 KO hearts).

To investigate whether a difference in the frequencies in living animals (7–8 Hz) or isolated cardiomyocytes (0.5 Hz) impacts myocellular Ca^2+^ signaling and contractile function, Ca^2+^ transient and SL shortening parameters were measured and subjected to a stimulation protocol. For this purpose, stimulation frequencies were stepwise increased from 0.5 to 2 Hz. Data collected at a stimulation frequency of 0.5 Hz were set to 100%. The peak amplitude of Ca^2+^ transients remained comparable between both groups when frequency was increased (Fig. 8*A*). In contrast, maximum SL shortening was increased by 23% at 2 Hz in KO compared to corresponding WT cardiomyocytes (Fig. 8*B*). The application of higher pacing frequencies was associated with an enhanced propensity to arrhythmias and irregular Ca^2+^ events in both genotypes. Arrhythmogenesis normally occurs during progressively incremented steady pacing frequencies leading to increased cytosolic Ca^2+^ levels that can activate an inward depolarizing NCX current. If sufficiently large, such transient inward currents could initiate proarrhythmic spontaneous delayed after depolarizations (16). Thus, we have measured Ca^2+^ transients and SL shortening only up to 2 Hz. An increase in stimulation frequencies had also an influence on the decay kinetics of Ca^2+^ transients. The half-maximum decay time was shortened from 1 to 2 Hz in KO compared to corresponding WT cardiomyocytes (Fig. 8*C*). The increase in frequency also resulted in an enhanced myocellular relaxation at 2 Hz in KO compared to corresponding WT mice (Fig. 8*D*).

## Discussion

This study investigated the functional role of PP2A-B56α in regulating cardiac Ca^2+^ cycling and contractility in response to β-adrenergic stimulation by use of a KO mouse model. We demonstrated that the deletion of B56α resulted in a lower basal PP2A activity and a compensatory increase in the expression of B′ subunits β and γ in the myocardium. Furthermore, this study showed that (i) the reduced basal PP2A activity was directly linked to an increased phosphorylation of Ca^2+^ regulatory and myofilament proteins in KO hearts. These changes were associated with an improved contraction and relaxation in catheterized mice under basal conditions. (ii) At the single cell level, in contrast, loss of B56α was associated with impaired Ca^2+^ transient characteristics and contractile parameters at basal conditions that were normalized by increasing stimulation frequencies. (iii) Ca^2+^ transients were comparable after β-adrenergic stimulation between both groups reflected by an unchanged PP2A activity and a normalized myocellular contraction and relengthening. (iv) Moreover, the impaired basal current density and inactivation kinetics of *I*_CaL_ in KO cardiomyocytes were restored by application of catecholamines, which is paralleled by higher phosphorylation levels of LTCC at Ser^1927^.

### Changes in the interplay between PP2A-Cα and its regulatory subunits in KO hearts

If, as previously shown (17), B56α has an inhibitory effect on PP2A activity, the deletion of B56α should result in an increase in activity. However, the loss of B56α was accompanied by a decreased activity of PP2A, which is most likely due to a lower expression of the catalytic subunit. These findings are opposite to the increased expression and activity of PP2A-Cα in cardiac-specific B56α-overexpressing mice (11). It seems that the expression levels of both regulatory and catalytic subunit adjust to each other to keep PP2A activity on a constant level. Proportionally related expression levels of B56α and Cα were also reported by other pathophysiological studies, such as in an endotoxemia model (18). At present, it is not clear whether B56α or other regulatory subunits can influence the expression of PP2A-Cα. The expression of PP2A-Cα is controlled by various transcription factors (19, 20). For example, a cAMP response element–binding site was localized in the Cα promoter (21) and inactivation of the cAMP-dependent transcription factor, CREB1, resulted in a decreased expression of PP2A-Cα (22). PP2A, in turn, is able to dephosphorylate CREB at Ser^133^ causing a mechanism of autoregulation of Cα expression (23). Thus, an altered phosphorylation of CREB, due to the KO of B56α followed by an impaired targeting of the PP2A holoenzyme, could well explain the reduced expression of the catalytic subunit. However, because CREB activity can also be increased by a PP2A-B56γ–mediated dephosphorylation at Ser^121^ (24), the increase in B56γ expression, detected in the present study, may represent a mechanism to normalize the decreased expression of PP2A-Cα. The increase in the expression of B56β and γ could itself be driven by PP2A activity (25, 26). It has already been shown in a cardiomyocyte-specific model that overexpression of rat B56γ increases PP2A activity (27). Furthermore, nuclear enrichment of B56γ resulted in an increased PP2A activity (28). Thus, our data suggest that the increased expression of B56β and γ may represent a functional compensation for the loss of B56α, since all 3 B′ subunits exhibit a large sequence homology and coincident localizations (2, 5, 29).

### Lower basal PP2A activity is associated with an enhanced phosphorylation of Ca^2+^ regulatory and contractile proteins and an improved contractility in catheterized KO mice

In the present study, intraventricular pressure and relaxation velocity were increased under basal conditions and after administration of intermediate doses of ISO in catheterized B56α KO mice. How does the decrease in PP2A activity links to these functional contractile effects? The answer to this question is primarily aimed on the molecular basis of these effects. Here, consistent with the *in vivo* contractile data, phosphorylation of PLN at Ser^17^, of troponin inhibitor at Ser^23/24^ and of MLC2 was increased under basal conditions in KO hearts that were excised from anaesthetized living mice. Thus, the decreased basal PP2A activity in KO hearts correlates well with the phosphorylation status of these key Ca^2+^ regulatory and contractile proteins. However, the decrease in PP2A activity does not affect all phosphoproteins. For example, the phosphorylation of PLN at Ser^16^, of RyR2 at Ser^2808/2814^, and of MyBP-C at Ser^282^ is unchanged between both groups. It is conceivable that these findings are indicative of a limited targeting of PP2A to these proteins by deletion of B56α. Specifically for the SR Ca^2+^ release channel as well as the MyBP-C, an association with PP2A-B56α was shown to be of functional relevance (30, 31). Thus, if a reduction of the PP2A catalytic activity has such different effects on phosphorylation levels of myocardial proteins, other mechanisms must be responsible for the regulation of the PP2A holoenzyme. It has already been shown that PP2A-B56α redistributes from the cell membrane to the cytosol after application of ISO, indicating a targeting function of B56α in response to β-adrenergic stimulation (11, 32). Such a targeting to specific subcellular localizations in response to the activation of different intracellular pathways could also be adopted by other PP2A regulatory subunits upon loss of B56α supposing an overlapping substrate specificity for individual cardiac regulatory proteins, such as the LTCC. In our KO model, both B56β and γ expression levels were increased, which may explain why the phosphorylation of Cav1.2 at Ser^1927^ was unchanged under basal conditions but increased (*i.e*., decreased dephosphorylation) after β-adrenergic stimulation regardless of an unchanged PP2A activity. It has been demonstrated that both subunits of LTCC, α and β, are substrates of PP2A, and a direct interaction between LTCC and PP2A was reported (7). Moreover, it has been suggested that PP2A is the main protein phosphatase that dephosphorylates the PKA phosphorylation site of LTCC at Ser^1928^ (33). Consistently, *I*_CaL_ was increased in murine cardiomyocytes when PP2A was blocked (34). Corresponding effects on action potential duration and frequency generation are conceivable. Thus, we suggest that the attenuated increase in heart rate after administration of ISO in our KO model could be explained by a unique PP2A targeting due to a specific expression pattern of regulatory PP2A subunits. The increase in phosphorylation of PLN at Thr^17^ and of TnI at Ser^23/24^ observed in addition to Cav1.2 after application of ISO despite unchanged PP activities might indicate a very local regulation of PP2A-associated phosphoproteins but also does not exclude currently unknown secondary effects. However, it is conceivable that a protein kinase A–mediated phosphorylation of B56δ after β-adrenergic stimulation is responsible for the normalization of the PP2A activity in KO hearts (3, 35).

### Depressed basal myocellular Ca^2+^ cycling and contractility is improved by β-adrenergic stimulation in KO cardiomyocytes

To our surprise, at the single cell level, there was a diminished maximum SL shortening in KO that was accompanied by a decreased peak amplitude of Ca^2+^ transients. An increased myofilament Ca^2+^ sensitivity suggests a compensation for the observed contractile impairment in KO. In search of the cause of the reduced Ca^2+^ transients, we first studied the role of Ca^2+^ permeating LTCC. Indeed, we detected a reduced maximum current density of *I*_CaL_ in KO cardiomyocytes. The integrated triggering *I*_CaL_ was reduced in KO cardiomyocytes as well, although *I*_CaL_ inactivation was delayed under basal conditions. This decrease in trigger Ca^2+^ may explain the observed reduction in the Ca^2+^ transient amplitude in KO (36, 37). At the same time, the decrease in Ca^2+^ transients may explain the prolonged inactivation of LTCC in KO cardiomyocytes, which is Ca^2+^ dependent (38, 39), providing an important feedback mechanism that tries to compensate for the reduced Ca^2+^ influx in KO cardiomyocytes. Thus, the slightly increased SR Ca^2+^ load in KO could help to keep the SR Ca^2+^ release as close to normal as possible, since fractional SR Ca^2+^ release increases with increasing SR Ca^2+^ load (40). In contrast, after application of ISO, we detected a comparable SL shortening and peak amplitude of Ca^2+^ transients in both groups, offsetting the genotypic differences observed under basal conditions in KO. Consistently, we found an unchanged PP2A activity between KO and WT hearts. The improved functional effects after administration of catecholamines in KO cardiomyocytes were also reflected by a normalization of LTCC parameters. It remains to be elucidated whether the increased phosphorylation of Cav1.2 at Ser^1927^ under β-adrenergic stimulation may contribute to the normalization of the reduced current density of *I*_CaL_. In addition, under β-adrenergic stimulation and phosphorylation of the RyR2, FKBP12.6 dissociates from the channel, resulting in an increased sensitivity to Ca^2+^-induced activation (41). As a result, the open probability of the SR Ca^2+^ release channel increases, which is highlighted by a higher ISO-induced Ca^2+^ spark frequency in KO cells in our study. This effect may contribute to overcome the impaired myocellular Ca^2+^ handling observed under basal conditions in KO.

### Enhanced inotropic and lusitropic effects in response to increasing stimulation frequencies in KO cardiomyocytes

To investigate whether differences in frequencies between living mice and isolated cardiomyocytes are responsible for the inconsistent contractile data, cells were subjected to increasing pacing frequencies. An increase in SL shortening and an accelerated relaxation and [Ca]_i_ decay has been expected for WT cells and was also observed in rat and rabbit cardiomyocytes (42, 43). However, the positive inotropic and lusitropic effects and the improved Ca^2+^ cycling kinetics under higher stimulation frequencies were more enhanced in KO than in WT cardiomyocytes. It is conceivable that the decreased PP2A activity in KO may contribute to a higher phosphorylation of regulatory proteins, as shown for PLN, TnI, MLC2, and MyBP-C (44, 45, 46), compared to WT cells. In addition, it remains to be elucidated whether an omitted or misdirected targeting of PP2A by loss of B56α is responsible for the improved contractile response to increasing stimulation frequencies in KO. The unchanged basal heart rate in catheterized living animals compared with the lower spontaneous beating rate in isolated Langendorff-perfused hearts (Fig. S5) of KO mice could be due to an increased sympathetic tone. We suggest that removal of the hearts reveals a basal sympathetic activation in KO mice because application of 1 μM ISO restored the chronotropic incompetence (Fig. S5). Evidence for a sensitization by catecholamines in KO mice can also be derived from the enhanced effects on the peak amplitude of Ca^2+^ transients and maximum SL shortening after application of ISO in cardiomyocytes.

### Limitations

PP2A and B56α are ubiquitously expressed proteins (5). In the present study, a global deletion of B56α was generated in our mouse model. Thus, the effects of a potentially altered PP2A activity in other tissues (*e.g*., vascular smooth muscle cells) on the heart are largely unknown. However, our initial hemodynamic studies in an iCre-induced heart-specific B56α KO model (Fig. S6) also showed an increased contractility and relaxation as observed in the global KO mice. Crossing transgenic mice with cardiac-specific overexpression of individual regulatory B′ subunits with our B56α KO model might reveal whether activity and targeting of PP2A could be restored. Because of the difficulty of obtaining sufficient sample material from isolated cardiomyocytes, analysis of the phosphorylation level of cardiac regulatory proteins under basal conditions, after administration of ISO and under different stimulation frequencies was not possible. However, these measurements are indispensable in future studies to investigate the influence of frequency on PP2A targeting and substrate specificity.

## Conclusion

We have confirmed by use of a global B56α KO mouse model that the increase in protein expression of individual members of the regulatory B′ gene family might compensate for the loss of one of their isoforms to maintain PP2A activity as close as normal. We have also demonstrated that the reduction in basal PP2A activity in KO hearts is followed by an enhanced phosphorylation of SR Ca^2+^ regulatory and myofilament proteins underlining the fundamental role of B56α in targeting of PP2A to specific substrates. The increased phosphorylation of PLN, TnI, and MLC2 is associated with a higher intraventricular pressure and relaxation in intact mice but not in isolated cardiomyocytes. However, higher stimulation frequencies and the application of catecholamines were able to normalize the impaired functional effects in KO cells. Our data provide new insights into the complex regulation of PP2A activity in cardiac muscle. Further studies are necessarily required to identify the molecular targets of PP2A-B56α in response to β-adrenergic stimulation.

## Experimental procedures

### Ethics statement

The use of experimental animals in this study was approved by the animal welfare committee of the University of Münster and the LANUV, NRW, Germany (ID 84-02.04.2014.A485), which also conform to the NIH Guidelines for the Care and Use of Laboratory Animals.

### Generation of B56α KO mice

The PP2A-B56α targeting construct was designed as follows. The 1.2 kb left flanking region containing exon 6 together with intron sequences was PCR amplified from mouse genomic DNA using oligonucleotides B56α_FlBd1 and B56α_FlBr1 (Table S1) and subcloned. The 5.2 kb right flanking region containing exons 8 to 13 and genomic sequences was PCR amplified using oligonucleotides B56α_FlAd1 and B56α_FlAr1 (Table S1) and consequently subcloned. The 0.5 kb exon 7 genomic region together with intronic sequences was PCR amplified and subcloned using oligonucleotides B56α_ex7d1 and B56α_ex7r1 (Table S1). The exon 7 flanking LoxP site together with the *Eco*RV and *Mlu*I sites were introduced by PCR cloning with help of the oligonucleotide B56α_ex7r1. All individual clones were verified by sequencing and assembled into the final targeting construct. The pBluescript-based plasmid backbone together with the negative selection marker (thymidine kinase cassette and diphtheria toxin gene) was added to the left flanking region. The positive selection marker (neomycin cassette flanked by 2 FRT sites) and 1 LoxP site was cloned as *Eco*RI—*Bam*HI DNA fragment between left flanking region and 0.5 kb exon 7 genomic PCR clone. The schematic representation of the PP2A-B56α (*Ppp2r5a*) gene targeting strategy is presented in Figure S7. The whole sequence of the PP2A-B56α targeting construct is presented in Figure S9.

The B56α TALEN nucleases N4 and N5 (Table S2) were designed using the Golden Gate TALEN and TAL Effector Kit 2.0 (47), using TAL effector vectors (48).

CV19 ES cells (passage 13 [129Sv × C57BL/6J]) were expanded in Hepes-buffered Dulbecco’s modified Eagle’s medium supplemented with 15% fetal bovine serum (PAA), nonessential amino acids, L-glutamine, β-mercaptoethanol, 1000 U of recombinant leukemia inhibitory factor (Merck Millipore) per milliliter, and antibiotics (penicillin [100 U/ml]; streptomycin [100 μg/ml]). For electroporation, 2 × 10^7^ cells were resuspended in 0.8 ml Capecchi buffer (20 mM Hepes [pH 7.4], 173 mM NaCl, 5 mM KCl, 0.7 mM Na_2_HPO_4_, 6 mM dextrose, and 0.1 mM β-mercaptoethanol (49)). The targeting vector pPP2A-B56α_targ was linearized with *Not*I, and 100 μg of DNA was electroporated together with 15 μg of each corresponding monomer DNA construct of each TALEN B56α nuclease at 25 μF and 400 V in 0.8 mm electroporation cuvettes (Gene Pulser, Bio-Rad). After electroporation, cells were cultivated for 10 min at room temperature (RT) and plated onto 10 100 mm diameter culture dishes containing a gamma-irradiated monolayer of mouse primary G418-resistant fibroblast feeder cells. Thirty-two hours later, 350 μg of G418 (Invitrogen) per milliliter and 0.2 μM 2′-deoxy-2′-fluoro-β-D-arabinofuranosyl-5-iodouracil (Moravek Biochemicals and Radiochemicals) were added to the culture medium. The medium was replaced every day, and colonies were picked and analyzed 8 days after plating.

Positively targeted ES cell clones were analyzed using the Southern blot DNA method. Approximately 5 μg of genomic DNA was digested with *Bam*HI, fractionated on 0.8% agarose gels, and transferred to GeneScreen nylon membranes (NEN DuPont). The membranes were hybridized with a ^32^P-labeled 1.6 kb probe containing sequences 5′ to the targeted homology and washed with (final concentrations) 0.5× SSPE (1× SSPE is 0.18 M NaCl, 10 mM NaH_2_PO4, and 1 mM EDTA [pH 7.7]) and 0.5% SDS at 65 °C. After first screening, correctly targeted event was proved on DNAs isolated from positively targeted ES cells with *Eco*RI, *Eco*RV, *Bam*HI, and *Kpn*I digestion.

Correctly targeted ES cells from independent clones were injected into 3.5 day B6D2F1 blastocysts. Routinely, we are injecting 12 to 14 ES cells into 1 blastocele. After injection, blastocysts were kept in KSOM medium and subsequently transferred into the uteri of 2.5 day pseudopregnant CD-1 foster mice. The mice carried pups to term. Chimeras were identified by their agouti coat color contribution. For the germline transmission, high percentage male chimeras were crossed to the C57BL/6J female mice. Heterozygous agouti offsprings (*B56α_targ* ^*+/−*^) were confirmed by the Southern blot analysis and further were tested by PCR for the presence of the targeted allele. The neo cassette flanked by FRT sites was deleted from the locus after crossing with the Flp-deleter mice, which ubiquitously express Flp recombinase (50). Cre-mediated *B56α* exon 7 excision was performed *in vivo* by crossbreeding mice harboring the PGK-1 promoter driven Cre transgene (51), resulting in total heterozygous *B56α*-deficient mice (*B56α* ^*+/−*^, Supplementary Figures S7 and S8). Crossbreeding of heterozygous mice resulted in homozygous *B56α* KO mice (*B56α*^−/−^).

### Histological examination

For detection of collagen structures, Masson-Goldner stainings were utilized. For this purpose, heart preparations were taken by transverse tissue sectioning at the level of the middle of the ventricles and then post-fixed in Bouin solution o/N at RT. In this process, the picric acid led to denaturation of the proteins. The following day, the excess Bouin solution was rinsed off with water for 15 min. Then, the heart preparations were subjected to iron hematoxylin staining according to Weigert. This resulted in dark brown to black staining of the nuclei (52). This was followed by the Masson-Goldner staining. Subsequently, heart preparations were dehydrated and preserved with Roti-Histokitt II. For documentation purposes, the heart transverse sections were imaged and saved using NIS Elements AR software (Nikon Instruments). Fibrotic fractions were quantified by Image Pro Analyzer software (Media Cybernetics).

### Immunofluorescence staining

Isolated cardiomyocytes were fixed by incubation with 4% formalin for 8 min, washed with PBS, and incubated on polylysine-coated slides for 30 min. Then, adherent cells were permeabilized with 0.1% Tergitol 15-S-9 (Merck) in PBS for 10 min. Cells were then incubated for 1 h in a blocking solution (2% goat serum, 1% bovine serum albumin, and 0.1% Tween-20 in PBS) containing 100 μg/ml Fab fragments (goat antimouse IgG (H + L)-unconjugated, Dianova). After washing for 5 min in PBS, cells were treated 1 h with an antibody against B56α (1:50, B56α antibody (F-10): sc-271151 Santa Cruz) or PPP2CA (1:200, anti-PP2A catalytic α: 610555 BD Transduction Laboratories) in blocking solution. After subsequent washing (3 × 10 min in PBS), cells were incubated with a secondary antibody Alexa Fluor 594 (1:500, Alexa Fluor 594 goat antimouse IgG, Thermo Fisher scientific) in blocking solution for 1 h at RT. After washing with PBS, cells were fixed with fluorescence mounting medium (Dako Deutschland GmbH). Fluorescence was measured using a confocal laser scanning microscope (LSM 710, Carl Zeiss AG).

### Quantitative real-time PCR

Using the Direct-zol RNA MiniPrep Kit, RNA was isolated from 12 mg of powdered heart tissue and subjected to DNase-1 treatment. The individual steps were performed according to the manufacturer's protocol. RNA preparations were stored at −80 °C or kept on ice for immediate determination of concentration. Quantification of RNA was performed photometrically as a triplicate determination using the P300 nanophotometer at a wavelength of λ = 260 nm. In addition, the absorbance of the samples was determined at an *A*_230_ and *A*_280_. If the quotients of *A*_260/280_ were between 1.8 and 2 and *A*_260/230_ was above 2, the RNA was defined as pure.

Reverse transcription was used to transcribe the pure RNA into its complementary DNA (cDNA) strand. Here, cDNA synthesis was performed using the Transcriptor First Strand cDNA Synthesis kit according to the manufacturer's protocol in the Gene AMP PCR System 9700. The PCR program included a primer hybridization phase at 25 °C for 10 min, followed by an elongation phase at 50 °C for 60 min. Reverse transcription was stopped by heating at 85 °C for 5 min. At the end of the experiment, cDNA was diluted 1:5 with RNAse/DNAse-free ultrapure water. Either the diluted samples were stored at −20 °C or tempered to 4 °C for immediate analysis by quantitative real-time PCR.

Quantitative real-time PCR was performed according to the manufacturer's information of the LightCycler 480 SYBR Green I Master Kit used (excitation at 465 nm and emission at 510 nm). For each approach, 2 μl of cDNA sample and 18 μl of the PCR mixture were pipetted into a LightCycler 480 Multiwell Plate 96. The primers and sequences used for the analysis of the corresponding target genes are listed in Table S3. Subsequently, the sample mixtures were centrifuged at 100x*g* for 2 min. The PCR reaction, detection, as well as melting curve analysis of the cDNA samples were performed with the LightCycler 480 II real-time PCR system according to an adapted protocol. The concentration of the DNA sequences was determined using the LightCycler 480 software (Roche). Hypoxanthine phosphoribosyl transferase-1 (*Hprt1*) served as the reference gene. Statistical analysis of the real-time quantitative RT-PCR data in Figure S4 was performed by the ΔΔCt method using the relative expression software tool (REST© version 2.013). Statistical random analysis was performed with 10,000 iterations.

### Hemodynamics

Hemodynamic measurements were performed according to an approved protocol (53). Experimental animals were anesthetized with an i.p. dose of 400 mg/kg of a 2% solution of tribromoethanol and the left jugular vein was connected to a microinfusion pump *via* a polyethylene tube. For data acquisition, a 1.4 Fr pressure-volume catheter (model SPR-839) was inserted into the left ventricle. The catheter was optimally placed when sinusoidal pressure curves were recorded using the MPVS-400 system and LabChart software (ADInstruments). After a 10 min stabilization period, a 5 μM ISO solution was applied to the left jugular vein *via* the microinfusion pump at a flow rate of 0.002 ml/min. After each 3 min, the flow rate was gradually increased up to 0.1 ml/min. At the end of the experiment, animals were killed by a repeated dose of tribromoethanol, followed by cervical dislocation in deep anesthesia. Heart rates and left ventricular pressures and volumes were acquired and analyzed throughout the experiment. To estimate the potency of ISO in both genotypes, sigmoidal dose–response curves were fitted to the measured values using the least squares approximation.

### Cell isolation

Isolation of ventricular cardiomyocytes followed an approved protocol (54). First, experimental animals were killed by cervical dislocation and the heart was immediately removed. The cannulated heart was then attached to a modified Langendorff apparatus and retrogradely perfused for 5 min with the perfusion buffer at a flow rate of 2.5 ml/min. For another 7.25 min, digestion was performed with an enzyme solution containing Liberase DH. Thereafter, the aorta and atria were separated from the heart. The remaining heart was minced in a 5 ml enzyme stop solution. After an incubation period of 10 min, the sedimented pellet was transferred into 10 ml of enzyme stop solution, filtered using nylon gauze, and centrifuged at 500 rpm for 1 min. The cell pellet was then resuspended with 10 ml of perfusion buffer and the Ca^2+^ concentration of the cell suspension was raised to 50, 100, 200, 500, and 1000 μM in 5 min steps. Then, centrifugation was performed again for 1 min at 500 rpm, and the cell pellet was resuspended in perfusion buffer containing 1 mM CaCl_2_. Cardiomyocytes were kept at RT and used within 6 h of isolation.

### Ca^2+^ transients and SL shortening

The experimental procedure followed an adapted protocol (55). For the measurement of Ca^2+^ transients and SL shortening, 100 μl cell suspension was incubated with 4 μM Indo-1-AM for 10 min in a perfusion chamber mounted on an inverted microscope. Cardiomyocytes were perfused with Tyrode’s solution and stimulated at 0.5 Hz (MyoPacer, Ionoptix). Subsequently, at least 10 cells were examined, recording at least 10 Ca^2+^ transients per cell and the corresponding SL shortening. After addition of 1 μM ISO, the same cells were analyzed again when the maximum β-adrenergic effect was reached. Alternatively, cardiomyocytes were subjected to a stimulation protocol of 1, 1.5, and 2 Hz for 1 min each. During measurement, cardiomyocytes were excited at 340 nm, and fluorescence emission at 405 and 495 nm was recorded using the Myocyte Calcium and Contractility System from Ionoptix. The data were automatically fitted by the Ionwizard software (IonOptix). The ratio of fluorescence emission (405 nm/495 nm) was calculated and regarded as an index of cytosolic Ca^2+^ concentration. Simultaneously, cell shortening was recorded with a video camera (Myo Cam-S) and SL shortening was calculated by fast Fourier transformation using the Ionwizard software. In addition, the relation between the peak amplitude of Ca^2+^ transients and SL shortening was applied as a loop function, whereby the slope can be used as an indicator of myofilament Ca^2+^ sensitivity.

### Measurement of SR Ca^2+^ load

SR Ca^2+^ release by rapid caffeine application was performed according to a revised protocol (55). Cardiomyocytes were perfused with Tyrode’s solution and at least 10 Ca^2+^ transients were recorded at 0.5 Hz. After a 10 s pause in stimulation, cells were superfused with 10 mM caffeine for 45 s using a rapid application aid. Within a few seconds, the cardiomyocytes responded immediately with a complete SR Ca^2+^ release and increased contraction (56). Data were obtained and analyzed, whereby the SR Ca^2+^ content corresponded to the maximum amplitude of the caffeine-induced Ca^2+^ transient and the fractional release to the ratio of Ca^2+^ transient amplitude at prestimulation and caffeine-induced peak amplitude of Ca^2+^ transients.

### Detection of Ca^2+^ sparks

For detection of Ca^2+^ sparks, cardiomyocytes were incubated with 4 μM Fluo-4 in the perfusion chamber of a confocal microscope (LSM710; Zeiss). Excess dye was removed and cells were then perfused with Tyrode’s solution supplemented with 1 μM ISO. During a pause in stimulation, a line scan (X-T scan) was performed. This cell line was excited at 488 nm and the fluorescence emission was detected in the range 505 to 622 nm. Analysis and detection of Ca^2+^ sparks were performed using ImageJ software (https://imagej.nih.gov/ij/) and the Sparkmaster plugin (57). Ca^2+^ spark frequency (CaSpF) was specified as number of sparks per square micrometer per second. Ca^2+^ spark amplitude (ΔF/F0) was automatically generated by the Sparkmaster plugin of ImageJ.

### Characterization of LTCCs

Voltage-gated LTCCs (*I*_CaL_) (58, 59) were recorded in Ca^2+^ tolerant ventricular myocytes, presenting clear striations, rectangular shape, and no signs of arrhythmia. Measurements were conducted using the perforated (amphotericin B, 280 μM) whole cell patch-clamp method in voltage clamp configuration. All recordings were performed at RT (22 ± 1 °C). Borosilicate glass capillaries (GB150TF-8P, Science Products) were pulled to a resistance of 3.5 ± 1 MΩ. Data were acquired, filtered at 10 kHz using an EPC-800 amplifier, and sampled with an 18 bit A/D converter InstruTech ITC-18 under the control of the PatchMaster software (HEKA Elektronik). Series resistance was compensated for at least 60%. The bath solution contained (in mM) the following: 136 NaCl, 5.4 KCl, 1.8 CaCl_2_, 1 MgCl_2_, 5 Hepes, 0.33 NaH_2_PO_4_, 10 TEA-Cl, 0.1 BaCl_2_, and 10 glucose, pH 7.4. Pipettes were filled with a solution containing (in mM) the following: 120 CsCl, 1 MgCl_2_, 5 Na_2_ATP, 10 TEA-Cl, 10 EGTA, and 10 HEPES, pH 7.2. Voltage protocols were applied from a holding potential of −40 mV and consisted of 400 ms activation steps between −55 to +60 mV in 5 mV increments. These activation steps were followed by a 300 ms step to 0 mV to evaluate the remaining fraction of closed channels (inactivation step). *I*_CaL_ amplitude was calculated as peak minus steady state current at the end of each pulse, and it was normalized to membrane capacitance to obtain the current density (pA/pF). Current-voltage (IV) relations, voltage dependence of activation and inactivation were calculated as described before (58, 59). For acute β-adrenergic stimulation, ISO was topically applied using a gravity driven perfusion system with a flow rate of 0.5 ml/min. The stock solution of 1 mM ISO in 5 mM ascorbic acid was weekly prepared and kept in dark at 7 °C. Prior to application, ISO was diluted to a final concentration of 1 μM in the bath solution and after each hour this solution was prepared freshly. Cardiomyocytes were kept in normal bath solution with 1 mM CaCl_2_ at RT and were used for recordings for up to 6 h after isolation. A current-time integral was used to estimate the Ca^2+^ entry by *I*_CaL_. After application of the main pulse from −40 to 5 mV (basal) or 0 mV (ISO) 200 ms of the enclosed area over the curve were used for the quantification and the obtained values were normalized to the cell capacitance.

### Protein phosphatase assay

The preparation of [^32^P]-labeled phosphorylase *a* (Phos *a*) was performed according to the protocol of Kirchhefer *et al*. (17) and is based on an established protocol (60). In brief, with the use of phosphorylase kinase, radioactive orthophosphate was incorporated in AMP-dependent phosphorylase *b*. The product of the phosphorylation reaction was [^32^P]-labeled AMP-independent Phos *a*, which served as a substrate of PP1 and PP2A. First, samples to be tested were mixed with a buffer that contained EDTA resulting in complexation of free Ca^2+^ and Mg^2+^ ions, which completely inhibited the catalytic activity of protein phosphatases, except for PP1 and PP2A. The dilution of the samples was chosen to convert no more than 18% of the maximum releasable [^32^P]. Because of the excess of [^32^P]-labeled Phos *a* substrate, this ensured that the dephosphorylation reaction was linear and not dependent on the amount of substrate. The dephosphorylation reaction was performed for 20 min at 30 °C by adding 20 μl of Phos *a*. Subsequently, 20 μl of 50% trichloroacetic acid was added to the mixtures, 20 μl of ultrapure water to the total count mixtures as a substitute, and 30 μl of bovine serum albumin solution. The trichloroacetic acid addition caused protein precipitation, which stopped the reaction. The mixtures were centrifuged at 14,000×*g* and 4 °C for an additional 10 min after incubation on ice for 10 min. Thereafter, 50 μl of the supernatant was analyzed in the scintillation counter. The specific activity of the Phos *a* substrate used was calculated from the difference between the total counts and the background radioactivity. To distinguish between PP1 and PP2A, samples were incubated in the absence and presence of 3 nM okadaic acid. At this concentration, okadaic acid completely inhibits PP2A activity, leaving PP1 activity unaffected (61).

### SDS-PAGE and immunoblotting

For generation of heart tissue, mice were anesthetized with 1.5% isoflurane. Subsequently, 4 mg/kg ISO was applied i.p. for 15 min. Anesthesia was withdrawn by delivery of 100% oxygen, and animals were killed by cervical dislocation. Hearts were removed, snap frozen, pulverized, and stored at −80 °C for follow-up experiments. Ventricular homogenates were prepared from 30 mg of tissue powder that was suspended in 300 μl of a 10 mM NaHCO_3_ solution containing a protease and phosphatase inhibitor cocktail (Roche). The mixture was homogenized with 3 10 s pulses at an intensity amplitude of 60% using ultrasound (HTU Soni 130). Thereafter, proteins were denatured with 100 μl of 20% SDS. After incubation at RT for 20 min, the mixtures were centrifuged at 14,000x*g* for an additional 20 min. From the supernatant, 10 μl were removed for subsequent determination of concentration. The remaining supernatant lysate was stored at −80 °C until further use.

Depending on the protein to be analyzed, a volume equivalent to 50, 100, or 200 μg of protein was taken from the prepared mixtures (see previous text) that were supplemented with 55 μl of 2×-Laemmli buffer, made up to 110 μl with a mixture of 3 parts 10 mM NaHCO_3_ solution and 1 part 20% SDS. When phosphorylation sites of target proteins were examined, the batch was incubated at 30 °C for 10 min, otherwise at 95 °C for 10 min. Subsequently, gel pockets were loaded and samples were electrophoretically separated on 5% or 10% SDS polyacrylamide gels. Proteins were transferred to a nitrocellulose membrane. Blots were incubated with specific antibodies raised against the following proteins: B56α of PP2A (1:2000, Bethyl, aa 25–75), Aα of PP2A (1:500, Santa Cruz), Cα of PP2A (1:2000, PTG), B56β of PP2A (1:250, Bio-Techne), B56γ of PP2A (1:500, Abcam), B56δ of PP2A (1:2000, Bethyl), B56ε of PP2A (1:1000, Aviva Systems), Cα of PP1 (1:500, Bio-Techne), inhibitor-1 of PP1 (1:10,000, Abcam), inhibitor-2 of PP1 (1:1000, Bio-Techne), cTnI (1:1000, Cell Signaling), cTnI phospho-Ser^23/24^ (1:1000, Cell Signaling), cTnI phospho-Thr^142^ (1:1000, Signalway AB), cMyBP-C (1:1000, LSBio), cMyBP-C phospho-Ser^282^ (1:1000, Enzo), Cav1.2 (1:200, Alomone), Cav1.2 phospho-Ser^1927^ (1:500, Thermo Fisher), MLC2 (1:1000, Cell Signaling), MLC2 phospho-Ser^18^ (1:1000, Origene), PLN (1:1000, Millipore), PLN phospho-Ser^16^ (1:5000, Badrilla), PLN phospho-Thr^17^ (1:5000, Badrilla), RyR2 (1:1000) (62), RyR2 phopsho-Ser^2808^ (1:1000, Badrilla), RyR2 phospho-Ser^2814^ (1:5000, Badrilla), calsequestrin (1:1000) (62), SERCA2a (1:1000) (62), Triadin (1:1000, Cloud-Clone), Junctin (1:1000) (62), and NCX (1:1000, Swant). The amounts of bound antibodies were detected by use of secondary antibodies (ECL rabbit/goat IgG, horseradish peroxidase–linked whole antibody, GE Healthcare). Signals were visualized and quantified with the ECL plus detection system (Amersham ECL Plus, GE Healthcare) and the ChemiDoc XRS system, respectively. All antibodies used have been applied several times in previous studies where they were already established. Initial testing was performed using protein dependence to determine the optimal amount according to linearity. Phospho-antibody adjustment was performed on the protein to be determined, and protein expression was adjusted to calsequestrin (CSQ). In addition, 1 set of samples was available for detection of proteins in Figures 2 and 4 that was probed with the CSQ antibody and then used for both figures. The same CSQ blot is therefore shown in both figures. A control sample was loaded on each blot to control for normal variation between blots. The individual samples were adjusted to this control sample.

### Statistics

Results are shown as means ± SD (scatter dot plots with bar) or standard error (2-D line plots). Individual data points represent individual cardiomyocytes per heart (“n”) or individual mice/hearts (“N”). The statistical analysis was performed using the SigmaPlot software (Systat Software GmbH). Depending on the research question and the comparison groups, various statistical tests were used. If the calculation resulted in a *p* value ˂0.05, differences between groups were considered significant. The respective tests and significances are noted in the figures and tables.

## Data availability

All the data produced for this work are contained within the article and the supporting information.

## Supporting information

This article contains supporting information (Figs. S1–S9; Tables S1–S3).

## Conflict of interest

The authors declare that they have no conflicts of interest with the contents of this article.
