# Supplementary material for: Impaired myocellular Ca2+ cycling in protein phosphatase PP2A-B56α KO mice is normalized by β-adrenergic stimulation
Source: J Biol Chem. 2022 Aug 10;298(9):102362. doi: 10.1016/j.jbc.2022.102362 (PMC9478386; doi:10.1016/j.jbc.2022.102362)
Supplement: Supplemental Figures S1–S9 [file mmc1.pdf]

Figure S1

A

B

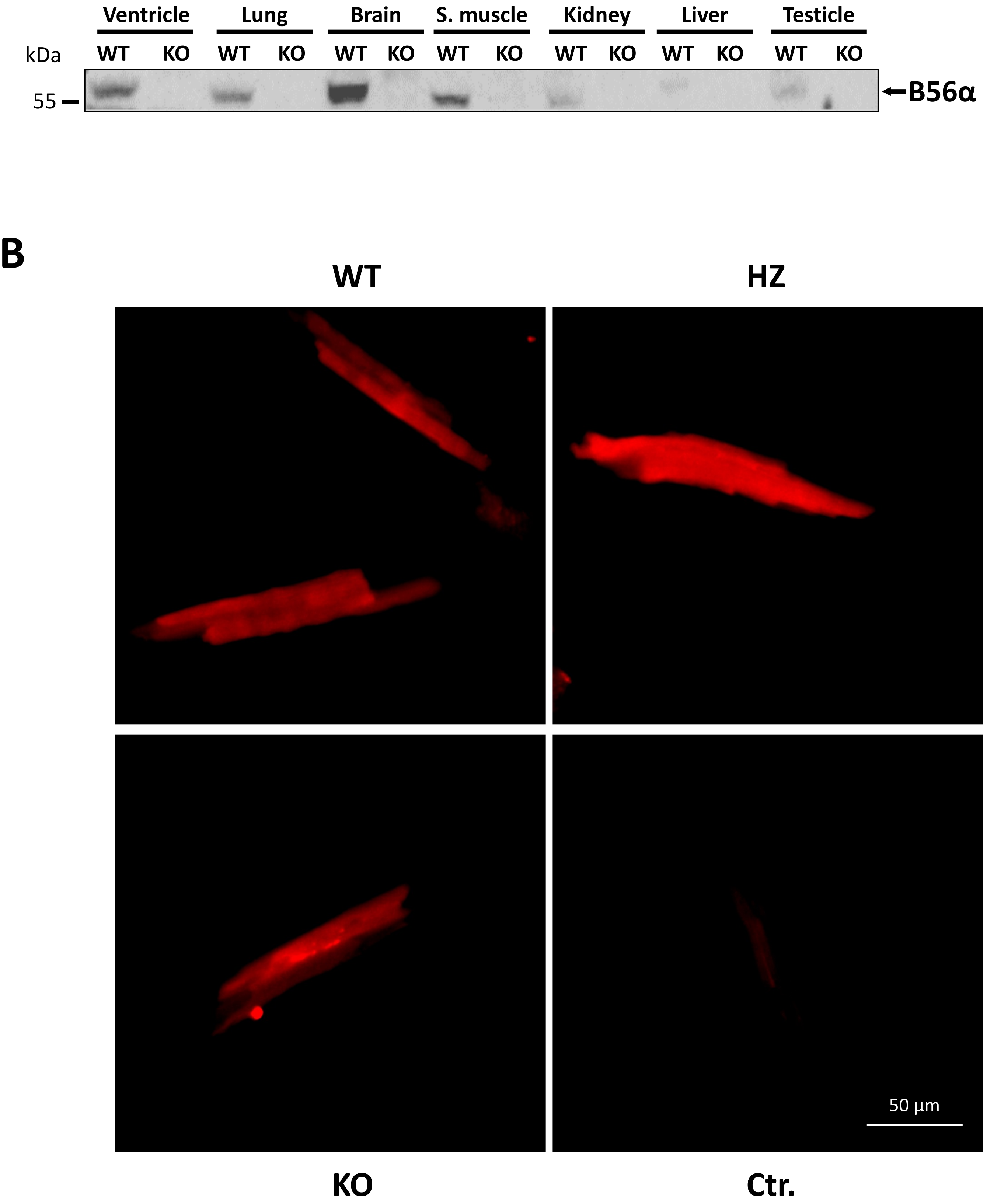

Figure S1. A: Protein expression of B56α in different tissues. Shown is an immunoblotting demonstrating the successful deletion of B56α. B: Photomicrographs showing detailed analysis of isolated cardiomyocytes from WT, HZ and KO mouse hearts by confocal microscopy. Red fluorescence staining represents the catalytic subunit α of PP2A. Control (Ctr.) was performed without the primary antibody.

Figure S2

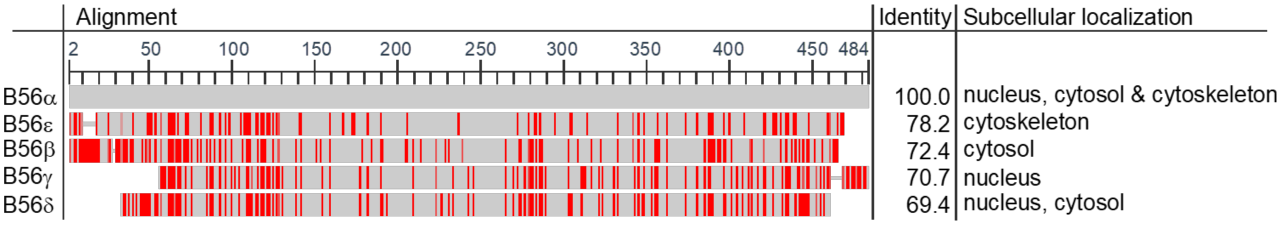

Figure S2. Sequence homology and subcellular localization of B' subunits of PP2A. Graphical representation of amino acid alignments of B' subunits sorted by identity using the NCBI Multiple Sequence Alignment Viewer 1.20.1. Different amino acids were highlighted in red.

Figure S3

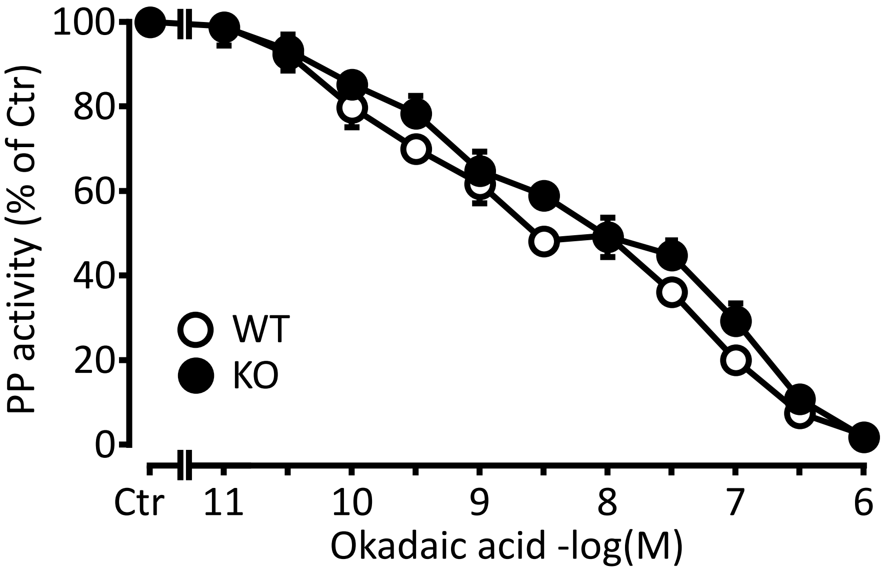

Figure S3. Protein phosphatase (PP) activity in ventricular homogenates. Graphical representation of PP activity in WT and KO hearts after inhibition with okadaic acid (N=3 hearts for each condition).

Figure S4

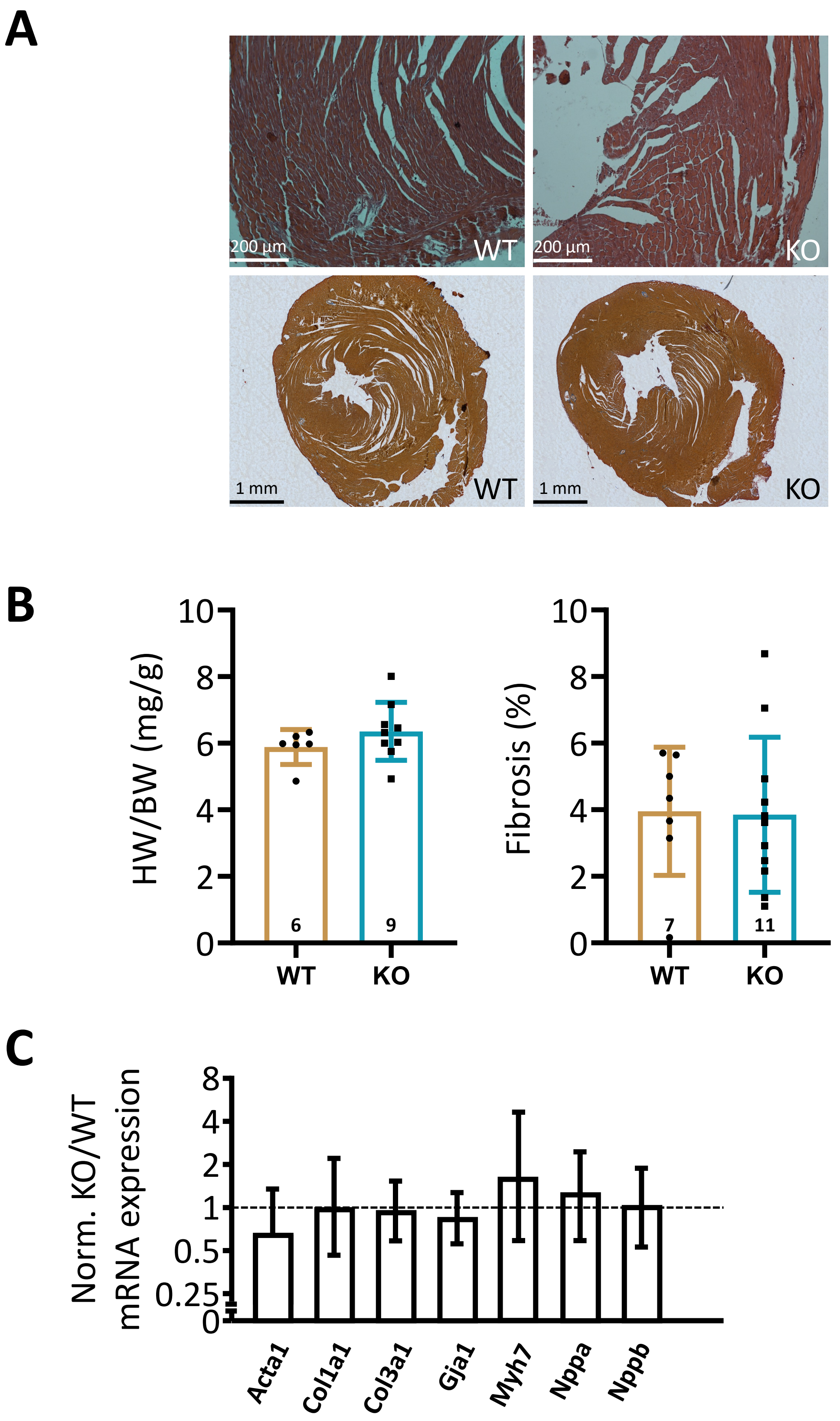

Figure S4. Unchanged heart morphology in KO mice. A: graphical representation of hematoxylin-eosin-stained ventricular sections (upper panel) and of connective tissue (blue-colored) using Masson-Goldner stainings (lower panel) in both genotypes. B: the ratio of heart to body weight (left panel) and the degree of collagen tissue (right panel) were determined (N=hearts). C: quantification of mRNA expression levels of marker proteins indicative for hypertrophy and fibrosis (Acta1, Actin alpha 1 skeletal muscle; Col1a1, collagen type I alpha 1 chain; Col3a1, collagen type III alpha 1 chain; Gja1, gap junction protein alpha 1; Myh7, myosin heavy chain beta (MHC-β) isoform; Nppa, natriuretic peptide A; Nppb, natriuretic peptide B) in WT and KO hearts. The mRNA expression levels were normalized to the reference gene HPRT. RT-PCR data was derived using and the relative expression software tool (REST© Version 2.013) and shown as relative mRNA levels ± SE normalized to WT and the house keeping gene Hprt (N=12 hearts for each condition).

Figure S5

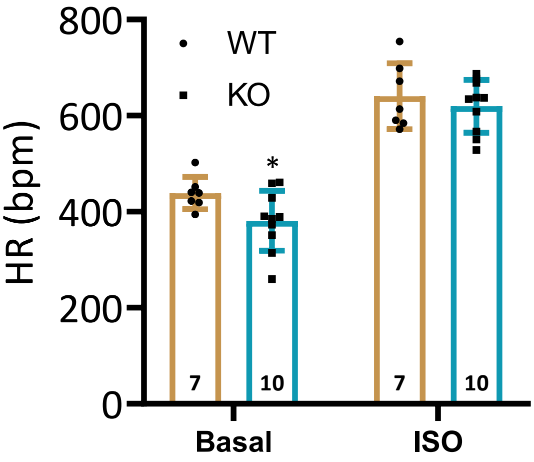

Figure S5. Beating rate in isolated Langendorff-perfused hearts. Heart rate (beats per minute) was measured under basal conditions (A) and after stimulation with 10^{-6} M isoprenaline (B) in WT and KO hearts (**P*<0.05 vs. WT; t-test; N=hearts).

Figure S6

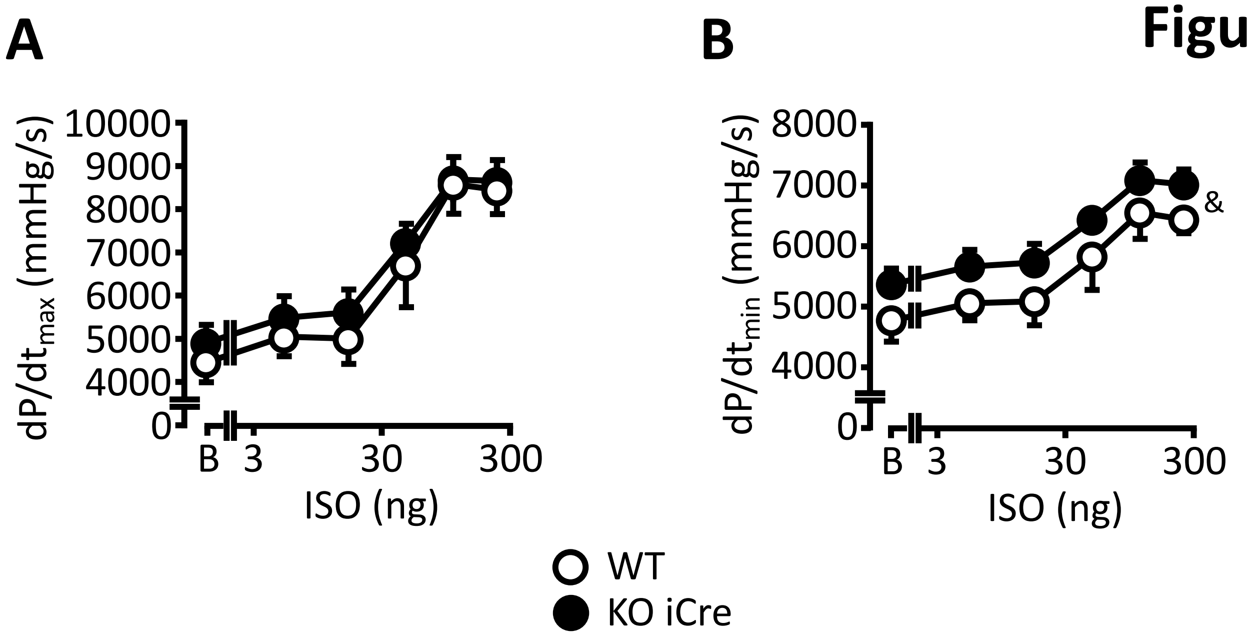

Figure S6. Shown are the contractile data in anaesthetized left-ventricular catheterized WT (floxed negative) and iCre-induced heart-specific KO mice in the absence and presence of increasing doses of isoprenaline (ISO). A: rate of contraction (dP/dt_{max}) in mmHg/s. B: rate of relaxation (dP/dt_{min}) in mmHg/s ($^{\&}P<0.05$ vs. WT: two-way ANOVA; N=6 WT mice; N=8 KO mice).

Figure S7

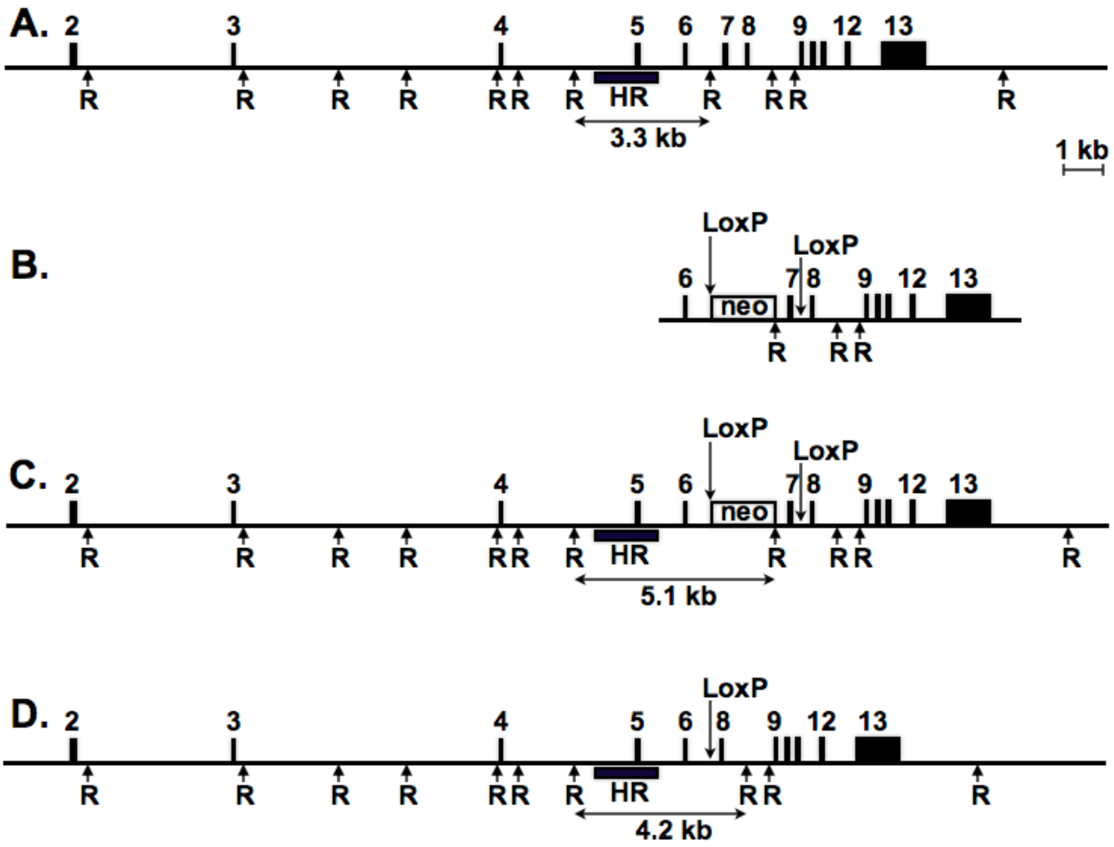

Figure S7. Schematic representation of the Pp2a-B56α (Ppp2r5a) gene targeting. Intronic and intergenic regions are shown as lines, and exons are represented as filled boxes numbered above. The empty box corresponds to the neomycin resistance cassette (neo). The vertical arrows indicate introduced LoxP sites. The arrows marked with "R" correspond to EcoRI restriction endonuclease sites. The black bars below (marked "HR") correspond to the sequence of hybridization probe used in Southern blot analysis. The expected sizes of restriction DNA fragments are labeled below. A: wild-type locus. B: targeted vector structure (without negative selection marker and plasmid backbone). C: genomic locus after the homologous recombination. D: genomic locus with eliminated exon 7 (KO) after crossing with the CRE recombinase expressing mice.

Figure S8

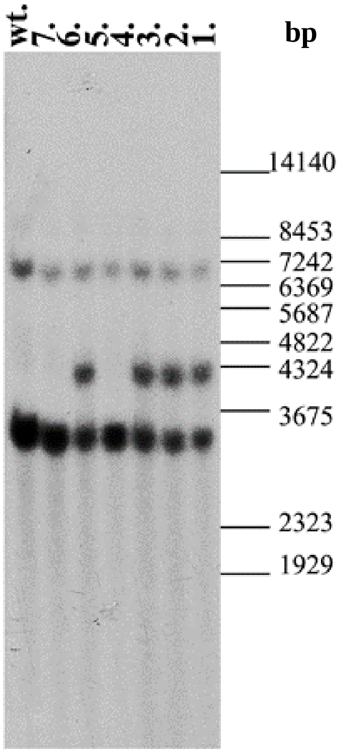

Figure S8. Southern blot analysis of genomic DNA (1-7) isolated from offspring obtained after crossing Pp2a-B56α gene targeted mice with the CRE recombinase expressing animal. The control wild-type (wt) DNA sample is presented on the top left. Positions of the size marker (in bp) are shown on the right. EcoRI enzymatic digestion reveals wild-type allele (3.3 kbp) and KO allele (4.2 kbp) fragments confirming heterozygous deletion of Pp2a-B56α exon 7 in DNA samples 1-3, and 6. The weak 6.8 kbp band corresponds to the region of the mouse chromosome 4, containing 134 bp sequence similarity to the HR probe.

Figure S9. Compilated sequence of the *PP2A-B56α* targeting vector (pPP2A-B56α _targ).

```
LOCUS       PP2A-B56α targeting construct 14119 bp    DNA     linear
FEATURES             Location/Qualifiers
     misc_feature    4564..5269
                     /note="RNA polII large subunit promoter"
     misc_feature    2114..3439
                     /note="TK gene"
     misc_feature    2332..3439
                     /note="Thymidine kinase"
     misc_feature    1967..2113
                     /note="HSV promoter/enhancer"
     misc_feature    complement(990..1929)
                     /note="ColE1 origin"
     misc_feature    129..990
                     /note="Ampicillin"
     misc_feature    3469..3522
                     /note="T2A"
     gene            3526..4182
                     /gene="DT-A"
     misc_feature    4241..4246
                     /note="polyA"
     misc_feature    5290..6444
                     /note="Flanking region B"
     exon            5895..5954
                     /note="exon 6"
     misc_recomb     complement(6508..6541)
                     /note="FRT"
     misc_recomb     6456..6489
                     /note="LoxP"
     polyA_signal    complement(6542..6844)
                     /note="bGHpolyA"
     CDS             complement(6858..7661)
                     /note="Neo"
     promoter        complement(7662..7727)
                     /note="EM7 promoter"
     promoter        7730..8244
                     /note="Pgk promoter"
     exon            8547..8655
                     /note="exon 7"
     misc_recomb     complement(8302..8335)
                     /note="FRT"
     misc_feature    8865..14025
                     /note="Flanking region A"
     exon            9179..9232
                     /note="exon 8"
     misc_recomb     8825..8858
                     /note="LoxP"
     exon            10493..10543
                     /note="exon 9"
     exon            10705..10824
                     /note="exon 10"
     exon            10908..11035
                     /note="exon 11"
     exon            11591..11692
                     /note="exon 12"
     exon            12457..13585
                     /note="exon 13"
ORIGIN
        1 GTGGCACTTT TCGGGGAAAT GTGCGCGGAA CCCCTATTTG TTTATTTTTC TAAATACATT
       61 CAAATATGTA TCCGCTCATG AGACAATAAC CCTGATAAAT GCTTCAATAA TATTGAAAAA
      121 GGAAGAGTAT GAGTATTCAA CATTTCCGTG TCGCCCTTAT TCCCTTTTTT GCGGCATTTT
      181 GCCTTCCTGT TTTTGCTCAC CCAGAAACGC TGGTGAAAGT AAAAGATGCT GAAGATCAGT
      241 TGGGTGCACG AGTGGGTTAC ATCGAACTGG ATCTCAACAG CGGTAAGATC CTTGAGAGTT
      301 TTCGCCCCGA AGAACGTTTT CCAATGATGA GCACTTTTAA AGTTCTGCTA TGTGGCGCGG
      361 TATTATCCCG TATTGACGCC GGGCAAGAGC AACTCGGTCG CCGCATACAC TATTCTCAGA
      421 ATGACTTGGT TGAGTACTCA CCAGTCACAG AAAAGCATCT TACGGATGGC ATGACAGTAA
      481 GAGAATTATG CAGTGCTGCC ATAACCATGA GTGATAACAC TGCGGCCAAC TTACTTCTGA
      541 CAACGATCGG AGGACCGAAG GAGCTAACCG CTTTTTTGCA CAACATGGGG GATCATGTAA
      601 CTCGCCTTGA TCGTTGGGAA CCGGAGCTGA ATGAAGCCAT ACCAAACGAC GAGCGTGACA
      661 CCACGATGCC TGTAGCAATG GCAACAACGT TGCGCAAACT ATTAACTGGC GAACTACTTA
      721 CTCTAGCTTC CCGGCAACAA TTAATAGACT GGATGGAGGC GGATAAAGTT GCAGGACCAC
      781 TTCTGCGCTC GGCCCTTCCG GCTGGCTGGT TTATTGCTGA TAAATCTGGA GCCGGTGAGC
      841 GTGGGTCTCG CGGTATCATT GCAGCACTGG GGCCAGATGG TAAGCCCTCC CGTATCGTAG
      901 TTATCTACAC GACGGGGAGT CAGGCAACTA TGGATGAACG AAATAGACAG ATCGCTGAGA
      961 TAGGTGCCTC ACTGATTAAG CATTGGTAAC TGTCAGACCA AGTTTACTCA TATATACTTT
     1021 AGATTGATTT AAAACTTCAT TTTTAATTTA AAAGGATCTA GGTGAAGATC CTTTTTGATA
```

1081 ATCTCATGAC CAAAATCCCT TAACGTGAGT TTTCGTTCCA CTGAGCGTCA GACCCCGTAG
1141 AAAAGATCAA AGGATCTTCT TGAGATCCTT TTTTTCTGCG CGTAATCTGC TGCTTGCAAA
1201 CAAAAAAACC ACCGCTACCA GCGGTGGTTT GTTTGCCGGA TCAAGAGCTA CCAACTCTTT
1261 TTCCGAAGGT AACTGGCTTC AGCAGAGCGC AGATACCAAA TACTGTCCTT CTAGTGTAGC
1321 CGTAGTTAGG CCACCACTTC AAGAACTCTG TAGCACCGCC TACATACCTC GCTCTGCTAA
1381 TCCTGTTACC AGTGGCTGCT GCCAGTGGCG ATAAGTCGTG TCTTACCGGG TTGGACTCAA
1441 GACGATAGTT ACCGGATAAG GCGCAGCGGT CGGGCTGAAC GGGGGGTTCG TGCACACAGC
1501 CCAGCTTGGA GCGAACGACC TACACCGAAC TGAGATACCT ACAGCGTGAG CTATGAGAAA
1561 GCGCCACGCT TCCCGAAGGG AGAAAGGCGG ACAGGTATCC GGTAAGCGGC AGGGTCGGAA
1621 CAGGAGAGCG CACGAGGGAG CTTCCAGGGG GAAACGCCTG GTATCTTTAT AGTCCTGTCG
1681 GGTTTCGCCA CCTCTGACTT GAGCGTCGAT TTTTGTGATG CTCGTCAGGG GGGCGGAGCC
1741 TATGGAAAAA CGCCAGCAAC GCGGCCTTTT TACGGTTCCT GGCCTTTTGC TGGCCTTTTG
1801 CTCACATGTT CTTTCCTGCG TTATCCCCTG ATTCTGTGGA TAACCGTATT ACCGCCTTTG
1861 AGTGAGCTGA TACCGCTCGC CGCAGCCGAA CGACCGAGCG CAGCGAGTCA GTGAGCGAGG
1921 AAGCGGAAGA GCGCCCAATA CGCAAACCGC CTCTCCCCGC gTCGAGGTCG AGCAGTGTGG
1981 TTTTCAAGAG GAAGCAAAAA GCCTCTCCAC CCAGGCCTGG AATGTTTCCA CCCAATGTCG
2041 AGCAGTGTGG TTTTGCAAGA GGAAGCAAAA AGCCTCTCCA CCCAGGCCTG GAATGTTTCC
2101 ACCCAATGTC GAGCAAACCC CGCCCAGCGT CTTGTCATTG GCGAATTCGA ACACGCAGAT
2161 GCAGTCGGGG CGGCGCGGTC CGAGGTCCAC TTCGCATATT AAGGTGACGC GTGTGGCCTC
2221 GAACACCGAG CGACCCTGCA GCGACCCGCT TAACAGCGTC AACAGCGTGC CGCAGATCTT
2281 GGTGGCGTGA AACTCCCGCA CCTCTTCGGC CAGCGCCTTG TAGAAGCGCG TATGGCTTCG
2341 TACCCCGGCC ATCAACACGC GTCTGCGTTC GACCAGGCTG CGCGTTCTCG CGGCCATAGC
2401 AACCGACGTA CGGCGTTGCG CCCTCGCCGG CAGCAAGAAG CCACGGAAGT CCGCCCGGAG
2461 CAGAAAATGC CCACGCTACT GCGGGTTTAT ATAGACGGTC CCCACGGGAT GGGGAAAACC
2521 ACCACCACGC AACTGCTGGT GGCCCTGGGT TCGCGCGACG ATATCGTCTA CGTACCCGAG
2581 CCGATGACTT ACTGGCGGGT GCTGGGGGCT TCCGAGACAA TCGCGAACAT CTACACCACA
2641 CAACACCGCC TCGACCAGGG TGAGATATCG GCCGGGGACG CGGCGGTGGT AATGACAAGC
2701 GCCCAGATAA CAATGGGCAT GCCTTATGCC GTGACCGACG CCGTTCTGGC TCCTCATATC
2761 GGGGGGGAGG CTGGGAGCTC ACATGCCCCG CCCCCGGCCC TCACCCTCAT CTTCGACCGC
2821 CATCCCATCG CCGCCCTCCT GTGCTACCCG GCCGCGCGGT ACCTTATGGG CAGCATGACC
2881 CCCCAGGCCG TGCTGGCGTT CGTGGCCCTC ATCCCGCCGA CCTTGCCCGG CACCAACATC
2941 GTGCTTGGGG CCCTTCCGGA GGACAGACAC ATCGACCGCC TGGCCAAACG CCAGCGCCCC
3001 GGCGAGCGGC TGGACCTGGC TATGCTGGCT GCGATTCGCC GCGTTTACGG GCTACTTGCC
3061 AATACGGTGC GGTATCTGCA GTGCGGCGGG TCGTGGCGGG AGGACTGGGG ACAGCTTTCG
3121 GGGACGGCCG TGCCGCCCCA GGGTGCCGAG CCCCAGAGCA ACGCGGGCCC ACGACCCCAT
3181 ATCGGGGACA CGTTATTTAC CCTGTTTCGG GCCCCCGAGT TGCTGGCCCC CAACGGCGAC
3241 CTGTATAACG TGTTTGCCTG GGCCTTGGAC GTCTTGGCCA AACGCCTCCG TTCCATGCAC
3301 GTCTTTATCC TGGATTACGA CCAATCGCCC GCCGGCTGCC GGGACGCCCT GCTGCAACTT
3361 ACCTCCGGGA TGGTCCAGAC CCACGTCACC ACCCCCGGCT CCATACCGAC GATATGCGAC
3421 CTGGCGCGCA CGTTTGCCCG GGAGATGGGG GAGGCTAACg gcggcggaga gggcagagga
3481 agtcttctaa catgcggtga cgtggaggag aatcccggcc ctaggatgga Ccctgatgat
3541 gtGgtGgatt cttctaaatc ttttgtgatg gaaaactttt cttcCtacca cgggactaaa
3601 cctggCtatg tGgattccat tcaGaaGggC atTcaGaagc caaaGtctgg CacacaGgga
3661 aattatgacg atgattggaa aggCttttat agCaccgaca ataaGtacga cgctgcTgga
3721 tactctgtGg ataatgaaaa cccTctctct ggaaaGgctg gaggcgtggt caaGgtgacT
3781 tatccaggac tgacTaaggt GctcgcactG aaagtggata atgccgaaac tattaagaaa
3841 gagCtGggCC tGagCctcac tgaaccTCtg atggagcaGg tcggaacCga agagtttatc
3901 aaGaggttcg gCgatggAgc ttcTcgCgtG gtgctcagcc tGccTttcgc tgagggCagC
3961 tctagcgtGg aataCatCaa taactgggaa caggcCaaGg cTCtGagcgt GgaactGgag
4021 attaatttg aaaccAgAgg aaaGAgAggc caGgatgcTa tgtatgagta tatggctcaG
4081 gcTtgtgcag gaaatAgAgt caggcgGtct ctCtgtgaag gaaccttGct Gctgtggtgt
4141 gacatCattg gacaGactac ctacagagat ttGaagctct aaAACACGGA AGGAGACAAT
4201 ACCGGAAGGA ACCCGCGCTA TGACGGCAAT AAAAAGACAG AATAAAACGC ACGGGTGTTG
4261 GGTCGTTTGT TCATAAACGC GGGGTTCGGT CCCAGGGCTG GCACTCTGTC GATACCCCAC
4321 CGAGACCCCA TTGGGGCCAA TACGCCCGCG TTTCTTCCTT TTCCCCACCC CACCCCCCAA
4381 GTTCGGGTGA AGGCCCAGGG CTCGCAGCCA ACGTCGGGGC GGCAGGCCCT GCCATAGCCA
4441 CTGGCCCCGT GGGTTAGGGA CGGGGTCCCC CtTGGGGAAT GGTTTATGGT TCGTGGGGGT
4501 TATTATTTTG GGCGTTGCGT GGGGTCTGGT GGACGACCCA GGAAGCTTTA AGGGTTCCGG
4561 ATCAGCTTGG GCTGAACATC GAGCGCCAGG GCTCCGTAAA GCTACTAGAG CACAGGCGGT
4621 GCCCCAACGT CCTGGGGCCT CTCCACTAAT AACGGCTACT TCCAATTGAT TGGACGCGCC
4681 ATCTTGCCTG CCTTATGCAT ATTCAGCGGT GAACTGAATA TTCATGAACG AGGCCCGTCC
4741 CGTCCCTCCC TCCTTCCCCC CACCCCCGGA ACCCGCTCCG GAGGACCCGA AGGGCCCCGC
4801 CTTCATTACC GATGCGTAGG ACAAACCATT TTCCCGATGT GTGTGGGGGG ATACTAATGA
4861 GAGACTTTAG CTGAAAATGA GCCTGAACTC CGAAGCTGAG TAAAAATGGC CTAACTTTAT
4921 CCTCCGTTCT GTAAGTCCTC GGTTTGAGTG CACGGGAAAC CCGAAAGGAG GACGACAGGA
4981 CCAGGAACAT TCTCCTCCTC CTGTCGCGTC AGAAAGAACA CCCAACCAGG GAGCCGGAGC
5041 CCTAGCGTCA ACAACTCCGC CGCGCGCGCT CCGTGTAGGC CGGTGCGGGC GGCCCCGTAG
5101 CGCAAGGGAG GGCGGGAAAG GAAGGGGCGG GACACAAGGG CGAATCTATA AAGGGCGTCA
5161 CTCAGCCAGT TCTCTCCTCA GAAGCGCCGA GAGCGCGACC GGGACGGTTG GAGAAGAAGG
5221 TGGCTCCCGG AAGGGGGAGA GACAAACTGC CGTAACCTCT GCCGTTCAGG ATCATCGAAT
5281 TCCTGTCGAC CTAGTCTTAA ATCAGTGTTC CATCTCCTCA GCTCTGCAGC CCCATGTCAC
5341 CCCCAACTCA TGCTGTCATT TGCTTTAAAG GCAGTTGTGA TAGTAGATAA CTAGATAGCC
5401 TATCAGTTAC TGTCTAAAGT TTCTGAACAG TTGAAAAAAT GGACCCCCTT TCCTCGGATT
5461 TTTATGTGAA TAGTGGTCTC CTACTTTGAA ACGGTTTCTT TAGGCAATCT GTCTTTCTTT
5521 TATATTAAGC GTAATATAAT GATCCCGGTG GAATAATACT ATGTGGCAGA TTTTAGCAGA
5581 GGACTGTGTG ACTCGGGAAA TAGATCCTGT GTTGTTACTG CTCTCAGTTT GGGCTGGGAT
5641 GTAGCTCATT TGGTGCTGTG GTGGCACACA CCTCTAGTGA CAACAGGGTC AGATGGTCAG
5701 GACTGGCTAC ACAGCAAGCT TAAAGGCAGT GTGGCAGTCT CAAAACAAAA CAGAGAGTCT

5761 GGTGTGAACC AGAAGCCTTG AGGCTGAGCA GCCACTGCTT GCTGTTACTG CTGCCTTGGG
5821 CTGAGTTTTT TCACCTTCCT GCCTTGTTCT TTGGTGTTTA CATTTTATTA CATCTATCTT
5881 TATTTCAAAT TCAGGTTTAT ATATGAAACG GAACATTTCA ATGGTGTGGC TGAGCTCCTG
5941 GAAATATTGG GAAGGTAGGT CAGGGGTGGG GTTTTGTTAT TTTTTAAGAA TTAAGTTACT
6001 GAAAGAGTAT TTTATATACA AAACAGCTGG AGTTGAAATG AGTTCCTATT TGAGAGTTTA
6061 CAGCACAATG CTAGATGCCC TGGGCTCAAT CCCTAACAgt gtgtgtgtgt gttgtgtgtg
6121 tgtgtgtgtg tgtgtgtgtg tgtATACACA CACACATACA CTGTTGTGTG TTGTTGGATC
6181 TACATAGCCT TGCCCCTTGG AAGTTCTTCC CAGCTGGAAG CCTGGAAGAG ATGAAGTTGT
6241 CATTCATTCA CTACAAAAAT TCTTACCCAa gccgggcagt ggtggcacaa ggctttaatc
6301 ccagcacttg ggaggcagaa gcagtcggaG GGGAGAGGGA GAGAGAGATC TTATCCAAAT
6361 GTCGATAGGC CATCCCCAAT CTAACTTTTC CTTTAGAAGA GTCTTCGGTT TTCTATACAC
6421 GTCCTTTCTG TTCACTACAC ACCAGGATCC ACCTAATAAC TTCGTATAGC ATACATTATA
6481 CGAAGTTATA TTATGTACCT GACTGATGAA GTTCCTATAC TTTCTAGAGA ATAGGAACTT
6541 CGAAGGGTTC CGCAAGCTCT AGTCGAGCCC CAGCTGGTTC TTTCCGCCTC AGAAGCCATA
6601 GAGCCCACCG CATCCCCAGC ATGCCTGCTA TTGTCTTCCC AATCCTCCCC CTTGCTGTCC
6661 TGCCCCACCC CACCCCCCAG AATAGAATGA CACCTACTCA GACAATGCGA TGCAATTTCC
6721 TCATTTTATT AGGAAAGGAC AGTGGGAGTG GCACCTTCCA GGGTCAAGGA AGGCACGGGG
6781 GAGGGGCAAA CAACAGATGG CTGGCAACTA GAAGGCACAG TCGAGGCTGA TCAGCGAGCT
6841 CTAGAGAATT GATCCCCTCA GAAGAACTCG TCAAGAAGGC GATAGAAGGC GATGCGCTGC
6901 GAATCGGGAG CGGCGATACC GTAAAGCACG AGGAAGCGGT CAGCCCATTC GCCGCCAAGC
6961 TCTTCAGCAA TATCACGGGT AGCCAACGCT ATGTCCTGAT AGCGGTCCGC CACACCCAGC
7021 CGGCCACAGT CGATGAATCC AGAAAAGCGG CCATTTTCCA CCATGATATT CGGCAAGCAG
7081 GCATCGCCAT GGGTCACGAC GAGATCATCG CCGTCGGGCA TGCGCGCCTT GAGCCTGGCG
7141 AACAGTTCGG CTGGCGCGAG CCCCTGATGC TCTTCGTCCA GATCATCCTG ATCGACAAGA
7201 CCGGCTTCCA TCCGAGTACG TGCTCGCTCG ATGCGATGTT TCGCTTGGTG GTCGAATGGG
7261 CAGGTAGCCG GATCAAGCGT ATGCAGCCGC CGCATTGCAT CAGCCATGAT GGATACTTTC
7321 TCGGCAGGAG CAAGGTGAGA TGACAGGAGA TCCTGCCCCG GCACTTCGCC CAATAGCAGC
7381 CAGTCCCTTC CCGCTTCAGT GACAACGTCG AGCACAGCTG CGCAAGGAAC GCCCGTCGTG
7441 GCCAGCCACG ATAGCCGCGC TGCCTCGTCC TGCAGTTCAT TCAGGGCACC GGACAGGTCG
7501 GTCTTGACAA AAAGAACCGG GCGCCCCTGC GCTGACAGCC GGAACACGGC GGCATCAGAG
7561 CAGCCGATTG TCTGTTGTGC CCAGTCATAG CCGAATAGCC TCTCCACCCA AGCGGCCGGA
7621 GAACCTGCGT GCAATCCATC TTGTTCAATG GCCGATCCCA TGGTTTAGTT CCTCACCTTG
7681 TCGTATTATA CTATGCCGAT ATACTATGCC GATGATTAAT TGTCAACAGG CTGCAGGTCG
7741 AAAGGCCCGG AGATGAGGAA GAGGAGAACA GCGCGGCAGA CGTGCGCTTT TGAAGCGTGC
7801 AGAATGCCGG GCCTCCGGAG GACCTTCGGG CGCCCGCCCC GCCCCTGAGC CCGCCCCTGA
7861 GCCCGCCCCC GGACCCACCC CTTCCCAGCC TCTGAGCCCA GAAAGCGAAG GAGCAAAGCT
7921 GCTATTGGCC GCTGCCCCAA AGGCCTACCC GCTTCCATTG CTCAGCGGTG CTGTCCATCT
7981 GCACGAGACT AGTGAGACGT GCTACTTCCA TTTGTCACGT CCTGCACGAC GCGAGCTGCG
8041 GGGCGGGGGG GAACTTCCTG ACTAGGGGAG GAGTAGAAGG TGGCGCGAAG GGGCCACCAA
8101 AGAACGGAGC CGGTTGGCGC CTACCGGTGG ATGTGGAATG TGTGCGAGGC CAGAGGCCAC
8161 TTGTGTAGCG CCAAGTGCCC AGCGGGGCTG CTAAAGCGCA TGCTCCAGAC TGCCTTGGGA
8221 AAAGCGCCTC CCCTACCCGG TAGAATTTCG ACGACCTGCA GCCAAGCTAG CTTGGCTGGA
8281 CGTAAACTCC TCTTCAGACC TGAAGTTCCT ATACTTTCTA GAGAATAGGA ACTTCGGAAT
8341 TCTGAGACAG TCACTCCTGT TGCTTTAAGG AGGGAAATTA AAGGCAGCTG TGCAGAACTC
8401 TGTAGTGACA GTGTAGATGT CAGATTCCAG GCTGCCCTGG AGGGACAGCT CAGCAGGCTG
8461 CCCTACCACT CCCCAGTGCT GTAGGTCATG TAACTTGTGG AAGCAAAGTG ACTACTTAAC
8521 CCTTCCCTGC CTTTTCTTCC CTCCAGTATC ATCAATGGCT TTGCATTGCC ACTGAAAGCA
8581 GAGCATAAGC AGTTTCTAAT GAAGGTTCTT ATTCCTATGC ATACTGCAAA AGGATTGGCC
8641 TTGTTTCACG CACAGGTAAA TTTCAGAACA ATTACAAATG GATATTTCCA AAAACAAATA
8701 TAAAGGCAAA AAAACTGAGA GGTATTAAGG TGCTAGTTTC TTGTAAAATT AAAGAATGTG
8761 CTTTGGCAAA ATTTTTATTA TGAGTTTGTT TACATACTAC TACTGCGGTT TTAACTTAga
8821 tatcATAACT TCGTATAGCA TACATTATAC GAAGTTATac gcgtGTTTGC TTCTTATATT
8881 TAGGTCTGGG AAGTGCTTAG TTGTGTGACC CTGAATGAAT CTCACAGGGA TGCAGAATGA
8941 TGCTAATGAT CTGTTAGGTC TTGGTCGCTG TTAACATTAG CACTGGAAGA CTAAATGGGT
9001 GTTTTTAAGT TAATTAAGTC TAGAGCTAAC AATATGTTGA ACTTAGAACT TTAATAAGAA
9061 TATATTCATC TTATACTGCA ACTTAAAACT GGAGTGACAT TTTGTGTCAG TGTGAAACTA
9121 TCCCCTTACC CCTAGTGTTA CGTCACTAAC ATCTCTTAAA CTTGCCTCTG CCCGTTAGCT
9181 GGCGTACTGT GTTGTGCAGT TCCTGGAGAA AGACACAACG CTGACAGAGC CGGTGAGTGT
9241 TCAGTTCCTG TTCATGCGCT TGTCTGGCCA CTGGTGTTTC ATTGCAGCCT GGGTAGTCAG
9301 TTCTCACACA GAGAGCAGCT TCTGGGAGCC TGAGAACGGA GCATGCTCAG GAGGCAGGAG
9361 GCATAAACTG AGGGACTGAT GGTTAAGATG TAGGTAAGAT CCAGAGACAA CGGTGAACTC
9421 TTAGCTCTCC TCACTCAATC CTTCTGTCAC TTCTTACGGT GAGAACACAC CGAGTCCCAG
9481 AACCATTCCC TTGAGTTTTG TTCAGAAATA AAAAGATTAG AGGTGTTTCC TCCCCTCCAG
9541 CAGTTTGGAG TTCAGTCTTA GAAATACTTA GTCATTGAGA AAAATGATTA AGATGAGCCG
9601 GTGTGAGAGC AGAAGGCTCA GCAGCTCAGC CCTAATGAAc tctgtaacct ttagcctggc
9661 tgatctagaa ctgtatttag cctagactgg cctcaaactt gtgatcctcc tgcctcagcc
9721 tcctaaatgc tgggatttTG AGCTCACCCA ACTCACTATA CAAACCACTG ATTGTACCCT
9781 TAAGTAAAAT GAGTGTTTCG GTAGAATGCT TTTTAAGCAC TTCAAATATA AGTACTGAAA
9841 CTGAAGGACT AGAATTCATG TATTTAGGCT AACCATAGGG GTGCTTGAAA TTTGATATGC
9901 ATGGCCTAAT TGTTGAAATA GATGGTCTCC TTTTAACATC TCAGTTTGCC CTGATTTTGT
9961 CAGCCATAAA ACAGGTTCGG GCACTCTAGA TAATGAGAAT TATCTGTTAG CTGTGATgag
10021 ctgaggcttg agcccagttg gtagggtgct ggcctagcat gcaccgggcc ctgggttcaa
10081 ccaccagtta cttatacggt cttggaaaaa caaaaaTGTT CTCGAAGATT AACTTCCTTC
10141 ACACCAGACA TCATTTTCTT GTTCTTTAAG TGTCCACTCA CGTTGGTCCC GTTCCATGAA
10201 GACCACAGAC TTTGCCCAGC CGTGTAGTAC CTAGAGCAGT GTCTGGCACG GCCCAGGTTC
10261 AAGTCCACAG CTAAGTAGGG ATTTTCATGT ACTGAGTGCT AAATGCTTGA CGTCAGGATG
10321 AGTGAAATAT CTTGGGATAG TCTAGCTAGT AGAAGTGGTa aggtctggct gtatcccagg
10381 ctggtctaga acttggaatt ctcctgcctg ggtacctcca agtgctagga ttgtagacgt

```
10441 gtaccacaca cccagttGTT GAATTCATTC TAAACTTTGC TGGTTTTTGT AGGTGATCAG
10501 AGGACTGCTG AAATTTTGGC CAAAAACATG CAGTCAGAAA GAGGTGGGtt ttaataataa
10561 taaaaataaa tGTATTTACA TTAGGACTGC AATCTTGACC TGCTCTGTGA ACTGTACCCA
10621 TTTCCTAATG TACCGATTTG CCTTGTCTAC ATTAGCATCA TTTGtaaatt ttttaaaagg
10681 aaaaaaaatt ttttttaaaC CTAGGTGATG TTTTTAGGAG AAATTGAAGA GATCTTAGAT
10741 GTCATTGAAC CAACACAATT CAAAAAAATT GAAGAGCCGC TTTTTAAGCA GATATCCAAG
10801 TGCGTCTCCA GCTCTCATTT CCAGGTACCA GCTTCCTTTC TCTGTGACAT TCTGTTGTTG
10861 TGCCATCCTT TTTCCTGCGG ATTTTGAGGT TTCTGGTGTC TTTTTAGGTT GCAGAAAGAG
10921 CACTGTACTT CTGGAATAAT GAATATATTC TTAGTTTGAT TGAAGAGAAT ATTGATAAAA
10981 TCCTGCCAAT CATGTTTGCC AGCTTGTATA AAATTTCCAA AGAACACTGG AATCAGTAAG
11041 TTTCTGTTGT TGTTGGGTTC CAtttaacat acagtctcag tagcacaggc tagccttgaa
11101 ctcttggctc agcctcagcg ctgagatcac aggcgtgtac ttcacgTGTC TTTACTTTGC
11161 TAGTTGAAGC CTGGACAGAT CTGTAACCTA TACTGAAGGT GAGTGTCATA GTTGTAGATA
11221 CTGCTTATCC TTATAAAGCT GTCTATGGAT GCTCCTCAGC AAATGGTCTA ACCAGCTTGC
11281 GGTCCAGTGC CATCGGCTGA GCCAGCTGTA TCCGGCCTCC CCAAGCTAGG AACTGAGGAC
11341 CAAAGGGAGA CATGGCATTC AGACATTCCG ACTTCTGTGA CATTTTTATC TTGCTACCCA
11401 GGGTCTAGGA TGAGACTACA AAACTCACAT GAAGAGAACC AGAATATTGT ATCTACATAG
11461 GAAGAGGGTA AACATTAGAA TCTCTGGCCT GTGAGACTAG ACTGTTTCCA CTTTAGTTCT
11521 AGCATCTGGC ATTAATTATG CATTCAGTGT TGCTGGCCTC TAACCCAGAA CTGTCTTCTC
11581 TTTCTTTCAG GACTATTGTA GCACTGGTGT ACAATGTGCT GAAAACCCTC ATGGAGATGA
11641 ACGGCAAGCT TTTTGACGAC CTTACTAGTT CCTACAAAGC CGAAAGACAG AGGTACTTGA
11701 TGAGTTTTGA AAATTCTGTG TTCTTCAGTT CAGAAAGGTC CATCTCTTAG TCGGTGGATC
11761 TATGGTGAAT ATGTTAGGCA TTATCATTTG GAAGCCTCTG AGCACTACAG CCATTGTCCA
11821 GGAAAGTAAT CCCGGTATTT TTATAAATTT GTTAAGACTT TCCTCCTCTA ACAGTTAGAC
11881 TGTGGTTTAA GTGCGGATCG TGTTCGTCCT GATCTCAGCT GAGACATAAG ATTTTTCCCG
11941 AGAGCTTGAT AGTCCTGGTG CTGTGGATCA TCAGAGAGTG GTATCTGAAG TCAGCACACT
12001 GTCAGGAAGC GAGTCAGAGC TCTGTGATTT TAAGGCCAGG AAGACACAAA CAAAAACCTC
12061 ACAAGGTTCA GGTAGAAATT AGAACCAGGA TCCAAGTCCA CAAAGCAACA GGCAGGAGAG
12121 GTGTGTGGGA ACCAAGACCT AGAAATAAAA GCCCTCCTTT ACAGCCTGTA CTTTCTTTAC
12181 TTATCTAGTT TTAAAGCTCA CTCAGTTTGA ACATAAACTG ACGGGTGGGT AGATTGCCTT
12241 TCTGCTGGTA TGGAAGGGTG GGCTTCTAC CTCTCCCTAG GAGGCCTGAG TGTTAACTGT
12301 AGCTGACCTA GAGGCCTCGA GTGACTACGG TGAATTAGTG CCATGTAATA CCACGCGTGT
12361 CACAGAAGCC ACGGGCATTG TAATGCTCGC TCCCCACAAA GCCACCAACC AGTGTCTTTC
12421 CTGAGCTTCC CATTAAAACT GCTGATCTCT CTACAGAGAG AAGAAGAAAG AACTGGAACG
12481 GGAAGAGTTG TGGAAAAAAC TAGAGGAGCT GCAGCTGAAG AAGGCTCTAG AGAAACAGAA
12541 CAATGCTTAC AACATGCACA GTATTCGCAG CAGTACCAGT GCCAAATAAA GATCAGCTCC
12601 CCCTGCTGGG CGGTCGTTTT GTACACTTTT TTTTTTTTTT GAATATATAA AAACTTCAGA
12661 GCAGACCTCA TCAGTATAAT ATAATTAGGA GGCCAGTTTT TCCTGGCAAG CGTAAAAGCG
12721 AAAGAATTAT GGACTAAAAC ATAGCCCTGT GCTGTATCAC GGCCACAGTA TATTGTAAAC
12781 TCTGTCTAAT CATGGATTGT GTCACTGTCT CTGTTGAGTG AGGTGATCGT GGGAGTGGCA
12841 AGCGTGTGTT GCGACTTGAG CCCGGTTGTG CTGCACACAC AGATGAAGCC GTCCTCTGCA
12901 CACTTCCTTT ATCATGTGTT TTCACCGTGC TGCACACCTT GGTGCTGCAC ACCTTGAGTA
12961 CATCTGAGGA AAGAGCCTCG TAAGATAAGC GGAGGGGTTG CCCTTCCCTC ACCTCTCCTA
13021 GAGAGGTGTG GGCAGGGGAC AAGAGCCCAG CCTCATTAAA GACACTGCCA TACTCTGGGT
13081 TTTACAACAT CTGACATTTC CAGGCTTCTG AAGCACAAAG TATTAAAGTT GGGGGGGGGA
13141 AGTAAACCAA AATTCTGATG TTCCCAAATC CCCCCTTCAG CAGCGGCTCC CCGGAGCGTG
13201 TGCGGAGCAG CACAGGCCAC GGGTGGACCC GAGGCTCACC TCCTTCATTC CTCTCCTCCA
13261 AGGCTGGAGG CAGGGCCTTC CCAGTCCTCA CCCTGCCAGT CCCCAGGCCT GCCTGCCTGC
13321 AGGGTGGAGC TCTGGGTCCC TCCCACAGTG TGATGCAGAC TGCTAGCTGT CACTGCCTGG
13381 CTTTATTTAA AGGAACTGCA GCAGGTGTCC TCAGAGCTGA CTATGTAGAA GCTTTGTCTG
13441 TTTTTACCTG GTCAGGTATT TTTCACACTG TTGTTACCAG TACCATTCCA GCCTCTTGCC
13501 TTGCAGTTGT ATGGAAAACT GTTTTATAAT GAGAGATCTT TACTGAGGAT TGAGCAGCAT
13561 TTAATAAAGT CTATGTTTGT ATTTTGCCTT ATGTCATTTG CTCTTTTGTC TCTGGAAGAT
13621 ATTTATATTA GAGAATTATA ACTTACGGAT GTACTCTAAT TAGAATTAGG TTGCTTGTGG
13681 TCCAGGGATT AGGAGTCTAG CCATCTAACC GATCTTTTCA TAAAATCCCA AGCTTCTTGT
13741 CCCTCCGTGT TCTAGGACAG TTTAACATTA AAGCTAGTTT TAACTAAAAC GGGAAATCTC
13801 AGAATCCATG GCAGTAGCAG TTCTGGGAAA GGCAGGGAGA CTGTAACATG GTTTCAACTA
13861 AACACCAGTT TAGAAGGGTC ACAGGGGTTT TTTGTTGCTG TGACAGCAGC CTCCATGACC
13921 TCTGGACAAA CTCCCTTGTA ATACAGTGGC CTGTAATTTT TGCTCTCTTT TCAAGCAGTG
13981 GGCATCCCAG GGAGGGCCAT TGTTGCAGTC CAATGAGGCT GCTCCgcggc cgcCGATTTC
14041 GGCCTATTGG TTAAAAAATG AGCTGATTTA ACAAAAATTT AACGCGAATT TTAACAAAAT
14101 ATTAACGCTT ACAATTTAG
//
```
