# Supplementary material for: Impaired myocellular Ca2+ cycling in protein phosphatase PP2A-B56α KO mice is normalized by β-adrenergic stimulation
Source: J Biol Chem. 2022 Aug 10;298(9):102362. doi: 10.1016/j.jbc.2022.102362 (PMC9478386; doi:10.1016/j.jbc.2022.102362)
Supplement: Supplemental Tables S1–S3 [file mmc2.pdf]

Table S1. List of the primers used.

Name	Sequence
B56a_FlAd1	TACGCGTGTTTGCTTCTTATATTTAGGTCTGG
B56a_FlAr1	TGCGGCCGCGGAGCAGCCTCATTGGACT
B56a_FlBd1	TGTCGACCTAGTCTTAAATCAGTGTTCCATC
B56a_FlBr1	TGGATCCTGGTGTGTAGTGAACAGAAAGGAC
B56a_ex7d1	TGAATTCTGAGACAGTCACTCCTGTTGCTTTA
B56a_ex7r1	TACGCGTATAACTTCGTATAATGTATGCTATACGAAGTTATGA TATCTAAGTTAAAACCGCAGTAGTAGTATGTA

Table S2. List of targets for the B56α TALEN nucleases.

Name	Left monomer DNA recognition sequence	Spacer sequence	Right monomer DNA recognition sequence
B56a_4	TCAGGGAAACCCCACCCC	CACCCCCCATTATCACTG	TGCATGCTGGGAATTCTA
B56a_5	TGTCAGGGAAACCCCACC	CCCACCCCCCATTATC	ACTGTGCATGCTGGGAA

Table S3. List of primers used in the real-time PCR.

Gene	Forward primer	Reverse primer
Acta1	CCGGGAGAAGATGACTCAAA	GAAGGAATAGCCACGCTCAG
Col1a1	CACCCTCAAGAGCCTGAGTC	GTTCGGGCTGATGTACCAGT
Col3a1	GTCCACGAGGTGACAAAGGT	GATGCCCACTTGTTCCATCT
Gja1	ACAGCGGTTGAGTCAGCTTG	GAGAGATGGGGAAGGACTTGT
Hprt1	ATGAGCGCAAGTTGAATCTG	GGACGCAGCAACTGACATT
Myh7	CTCCCAAGGAGAGACGACTG	TTAAGCAGGTCGGCTGAGTT
Nppa	GGGGGTAGGATTGACAGGAT	CAGAGTGGGAGAGGCAAGAC
Nppb	GACCAAGGCCTCACAAAAGA	AGACCCAGGCAGAGTCAGAA
